# Characterizing and Predicting Autism Spectrum Disorder by Performing Resting-State Functional Network Community Pattern Analysis

**DOI:** 10.3389/fnhum.2019.00203

**Published:** 2019-06-14

**Authors:** Yuqing Song, Thomas Martial Epalle, Hu Lu

**Affiliations:** School of Computer Science and Telecommunication Engineering, Jiangsu University, Zhenjiang, China

**Keywords:** autism spectrum disorder, resting-state connectivity analysis, community detection, machine learning, linear discriminant analysis

## Abstract

Growing evidence indicates that autism spectrum disorder (ASD) is a neuropsychological disconnection syndrome that can be analyzed using various complex network metrics used as pathology biomarkers. Recently, community detection and analysis rooted in the complex network and graph theories have been introduced to investigate the changes in resting-state functional network community structure under neurological pathologies. However, the potential of hidden patterns in the modular organization of networks derived from resting-state functional magnetic resonance imaging to predict brain pathology has never been investigated. In this study, we present a novel analysis technique to identify alterations in community patterns in functional networks under ASD. In addition, we design machine learning classifiers to predict the clinical class of patients with ASD and controls by using only community pattern quality metrics as features. Analyses conducted on six publicly available datasets from 235 subjects, including patients with ASD and age-matched controls revealed that the modular structure is significantly disturbed in patients with ASD. Machine learning algorithms showed that the predictive power of our five metrics is relatively high (~85.16% peak accuracy for in-site data and ~75.00% peak accuracy for multisite data). These results lend further credence to the dysconnectivity theory of this pathology.

## 1. Introduction

The study of the human brain often confronts problems arising from the brain's inherent complexity(Bullmore and Sporns, 2009). To overcome this challenge, complex network analysis methods have been extensively used in neurosciences, where the human brain is typically modeled as a network or graph whose nodes represent brain regions and edges represent the anatomical or functional interactions among them(De Vico Fallani et al., 2014). Network representation has been a promising computational model to capture the brain's topological organization as well as its dynamics(Rubinov and Sporns, 2010). Studies in this area have revealed that the human brain has a scale-free small-world topology(Eguíluz et al., 2005) with modular fragmentation and highly connected hubs(Meunier et al., 2010; Nicolini et al., 2017).

One problem eliciting interest in the analysis of resting-state functional brain networks by using complex network methods is community detection(Fortunato, 2010), which can be described as the unsupervised discovery of subgroups of brain regions that are typically activated together and densely connected(van den Heuvel et al., 2008; Shen et al., 2010). Several studies have shown that this modular structure of the functional network reflects the anatomical and functional segregation of the human brain, with the presence of hub nodes or regions sharing numerous inter-community edges. Recent studies have suggested that community hubs are highly vulnerable to the effects of brain disorders, resulting in an altered community structure observed in several neuropsychiatric pathologies(Nicolini et al., 2017).

Previous studies using complex networks methods to the study of neurological disorders aimed to characterize the differences between normal and pathological brains. Graph theoretical metrics illustrated alterations in the resting-state functional connectome under specific neurological pathologies, including trauma(van der Horn et al., 2017), amnestic mild cognitive impairment(Chen et al., 2012), Alzheimer's disease(Supekar et al., 2008), epilepsy(Ponten et al., 2007), attention deficit/hyperactivity disorder(ADHD)(Wang et al., 2009; Ahmadlou and Adeli, 2011), and autism spectrum disorder (ASD)(Zhou et al., 2014). In addition, machine learning techniques using different types of features have been increasingly used not only to detect pathology-related alterations but also to make individual subject predictions of brain disorders(Arbabshirani et al., 2017).

ASD is typically characterized by deficits in social interaction and communication, rigid and stereotypical behaviors, and abnormal sensory processing(Rapin and Tuchman, 2008). This neurological disorder has been classified as a dysconnectivity syndrome manifesting as the disruption or abnormal integration of brain regions evidenced by changes in network properties used as diagnostic markers(Hull et al., 2016). In the task of automatically detecting ASD by using resting-state functional MRI (rsfMRI) data, different types of features, including independent component analysis (ICA)(Uddin et al., 2011) and functional connectivity among regions of interest (ROIs)(Iidaka, 2015; Plitt et al., 2015), have been used in conjunction with various machine learning algorithms, such as logistic regression, random forest, and neural network algorithms.

In this study, we compared the resting-state functional community patterns of patients with ASD and controls at the group and individual level to gain a detailed understanding of the relationship between impaired connectivity and this brain pathology. We also reconstructed the communities of each ROI and used a permutation test based on the Rand index to detect the brain regions whose community structures differ significantly between patients with ASD and controls.

In previous studies applying network community pattern analysis to research brain disorders, modularity (a complex network metric) has emerged as a *de facto* standard to quantify the alterations in the distribution of inter-community vs. intra-community edges under a specific brain disorder. Despite the increasing popularity of this single metric in community detection approaches, one common drawback of single indices is their low sensitivity and specificity (Stam and van Straaten, 2012). Autism being a complex disorder, the underlying neural phenomenon could be better captured by combined community patterns indices beyond the individual capability of single metrics. Here, we used modularity as well as other descriptive community pattern metrics drawn from the complex networks literature that have not been previously used for analysing the community structure of resting-state functional connectivity networks built from neuroimaging data. By using experimental data from 235 subjects in six publicly available datasets and validation data from 214 subjects in six additional datasets, we showed that these five community pattern metrics alone can serve as efficient single-subject predictors of autism.

## 2. Materials and Methods

### 2.1. Datasets

Experimental data were selected from the Autism Brain Imaging Data Exchange (ABIDE), a large multisite, publicly available repository of resting-state fMRI scans, forming part of the 1000 Functional Connectomes Project (Di Martino et al., 2014). The data were downloaded from five sites: Stanford University (STA), University of Leuven Sample 1 (LV1), University of Leuven Sample 2 (LV2), Olin Institute of Living at Hartford Hospital (OLI), University of Pittsburgh, School of Medicine (PIT), and California Institute of Technology (CAL). The imaging data included technical scan parameters as well as phenotypic information of each individual. Demographic information about participants in each dataset is shown in Table 1; Table S1 provides the technical details of the scans. As part of the professional and ethical protocol of the 1000 Functional Connectomes Project, all datasets have been anonymized, and no protected health information was included. Despite the availability of phenotypic information, this study did not use any of this medical or biological information to analyse group differences or predict the clinical class of individual participants.

**Table 1:** Datasets.

	**ASD**		**Control**		**Age($\bar{x}\pm \sigma$)**	**Total**
**Dataset**	**M/F**	**Age**	**M/F**	**Age**		***N* = 235**
STA	16/4	7.5–12.9	16/4	7.8–12.4	9.9 ± 1.5	*n* = 40
LV1	15/0	18–32	14/0	18–32	22.5 ± 3.5	*n* = 29
LV2	12/3	12.1–16.8	15/5	12.2–16.9	14.16 ± 1.42	*n* = 35
OLI	17/3	11–24	14/2	10–23	16.8 ± 3.4	*n* = 36
PIT	26/4	9.3–35.2	23/4	9.4–33.2	18.9 ± 6.8	*n* = 57
CAL	15/4	17.5–45.1	15/4	17–56.2	28.15 ± 0.41	*n* = 38

*M, male; F, female; $\bar{x}$, mean; σ, standard deviation*.

### 2.2. Descriptive Community Pattern Metrics

In the last decade, community detection has become a prolific research area in complex networks and pattern recognition(Pons and Latapy, 2006; Fortunato, 2010; Epalle and Liu, 2016), with many application domains, such as social network mining(Girvan and Newman, 2002), graph visualization(Bastian et al., 2009), compression(Hernández and Navarro, 2012), parallel computing (Ngonmang et al., 2012), and recommender systems(Liben-Nowell and Kleinberg, 2007). In neuroscience, community detection has been applied as an important step in resolving more complex problems, such as localizing network alterations in specific brain disorder(Lerman-Sinkoff and Barch, 2016). In this subsection, we introduce the basic mathematical notations for community detection and review modularity as well as four other metrics used to describe community structure in graphs.

A network or graph *G* = (*V, E*) is composed of a set of nodes *V* and a set of edges *E*. In this study, the nodes *V*, representing brain regions, are labeled 1, 2, 3…, *N*, with *N* = 90. If an edge (*x, y*) is in *E*, then node *x* is connected to node *y*. If *G* is undirected and unweighted, the adjacency matrix *A* of *G* is the matrix of 0s and 1s such, that *A*_*xy*_ = 1 if and only if (*x, y*) ∈ *E*. Community detection, being a clustering of *G*, can be defined as a partition of *V* into the sets *V*_1_, …, *V*_*K*_ such that *V*_1_∪…∪*V*_*K*_ = *V*, and *V*_*i*_∩*V*_*j*_ = ∅ for any *i*≠*j*, with none of the *V*_*i*_ being empty. The sets *V*_1_, …*V*_*K*_ are called communities or clusters. Any partition **V** = {*V*_1_, …, *V*_*K*_} is a community structure or community pattern of a network with *K* = |**V**| communities.

Community patterns are commonly described in terms of quality functions, which depend on both the graph *G* and the partition **V** and whose optimization is typically believed to yield the best community pattern. However, these metrics can be considered as descriptive of a network's modular organization, rather than true performance metrics, because they do not provide strict quantitative criteria for more and less optimal partitioning(Steinhaeuser and Chawla, 2010).

In this study, we investigated the community organization of resting-state functional brain networks in ASD by using the following descriptive metrics:

#### 2.2.1. Modularity

Modularity (*Q*) is the most popular community characterization metric in the literature. In a network *G* = (*V, E*) and a partition **V** = {*V*_1_, …, *V*_*K*_}, the edges of *G* can be grouped into community bridge sets *B*_*ij*_ as follows: (*x, y*) ∈ *B*_*kl*_ if and only if *x* ∈ *V*_*k*_ and *y* ∈ *V*_*l*_. In particular, we note $B_{k}^{i}=B_{kk}$ as the set of internal edges of *V*_*k*_ having all their ends in the same community; we note $B_{k}^{e}=\cup_{k\neq l}B_{kl}$ as the set of external edges of *V*_*k*_ having one end in *V*_*k*_ and the other in **V**−*V*_*k*_. By using these notations, a network's modularity is defined as

(1) $$Q(G,V)=\sum_{k=1}^{K}\left(\frac{2|B_{k}^{i}|}{2m}-{\left(\frac{m_{k}}{2m}\right)}^{2}\right),$$

where $m_{k}=\underset{x\in V_{k}}{\sum }$$\underset{y\in V}{\sum }A_{xy}$ is the total degree of community *V*_*k*_ and *m* the total number of edges in the network.

The four other community pattern metrics which were first introduced in(Mitalidis et al., 2014) have so far received little attention from the scientific community probably because they were proposed after the publication of two authoritative review articles on complex network measures of brain connectivity(Bullmore and Sporns, 2009);(Rubinov and Sporns, 2010).

#### 2.2.2. Global Density

The global density community quality function (not to be confused with the popular density metric) is defined as

(2) $$Q_{GD}(G,V)=\frac{1}{2}[Q_{GD}^{i}(G,V)+1-Q_{GD}^{e}(G,V)],$$

where

$$Q_{GD}^{i}(G,V)=\frac{\sum_{k=1}^{K}\sum_{x\;\in \;V_{k}}\sum_{y\;\in \;V_{k}}A_{xy}}{\sum_{k=1}^{K}{\left|V_{k}\right|}^{2}}$$

represents the global internal density and

$$Q_{GD}^{e}(G,V)=\frac{\sum_{k=1}^{K}\sum_{x\;\in \;V_{k}}\sum_{y\;\in \;V-V_{k}}A_{xy}}{\sum_{k=1}^{K}\left|V_{k}|*|V-V_{k}\right|}$$

represents the global external density. This formula assumes that *A*_*xx*_ = 1 for all *x* ∈ *V*, and all other edges are counted twice. *Q*_*GD*_(*G*, **V**) takes values in [0,1], where the value 1 is assigned only to graphs with perfect community structure.

#### 2.2.3. Local Density

The local density quality function is defined as

(3) $$Q_{LD}(G,V)=\sum_{k=1}^{K}\frac{\left|V_{k}\right|}{2|V|}*[q^{i}(V_{k},G)+1-q^{e}(V_{k},G)],$$

where the local inner and outer densities are, respectively, defined as

$$q^{i}(V_{k},G)=\frac{\sum_{x\in \;V_{k}}\sum_{y\in \;V_{k}}A_{xy}}{|V_{k}{|}^{2}}$$

and

$$q^{e}(V_{k},G)=\frac{\sum_{x\in \;V_{k}}\sum_{y\in \;V-V_{k}}A_{xy}}{\left|V_{k}|*|V-V_{k}\right|}.$$

*Q*_*LD*_ is defined slightly differently than is *Q*_*GD*_, but both are based on the idea of communities being formed by subsets of nodes that are more densely connected with each other than externally. *Q*_*LD*_ also takes values in [0,1].

#### 2.2.4. Distance-Based Metric

The distance-based community quality function is defined as

(4) $$Q_{DB}(G,V)=\frac{1}{|V{|}^{2}}||A_{G}-A_{V}||,$$

where $||B||=\sum_{x\in \;V}\sum_{y\in \;V}\left|B_{xy}\right|$ is a matrix norm, *A*_*G*_ is the adjacency matrix of *G*, and *A*_*V*_*xy*__ = 1 if *x, y* belongs to the same cluster (under *V*), whereas *A*_*V*_*xy*__ = 0 if *x, y* belongs to different clusters (under *V*). *Q*_*DB*_ takes values in [0,1], but unlike with the other metrics, the value 0 is obtained for graphs exhibiting a perfect community structure.

#### 2.2.5. Node Membership Metric

The node membership community quality function is defined as follows:

(5) $$Q_{NM}(G,V)=\frac{1}{2|V|}\underset{x\in \;V}{\sum^{\text{​}}}[\mu (x,V[x])+1-\mu (x,V-V[x])].$$

*V*[*x*] indicates the cluster to which *x* belongs and node membership is defined by

$$\mu (x,U)=\frac{1}{\left|U\right|}\sum_{y\in \;U}A_{xy}.$$

Hence, μ(*x, U*) = 1 if and only if *x* is connected to every *y* ∈ *U* and μ(*x, U*) = 0 if and only if *x* is connected to no *y* ∈ *U*; for intermediate situations, we obtain μ(*x, U*) ∈ ]0, 1[.

**Figure 1:**
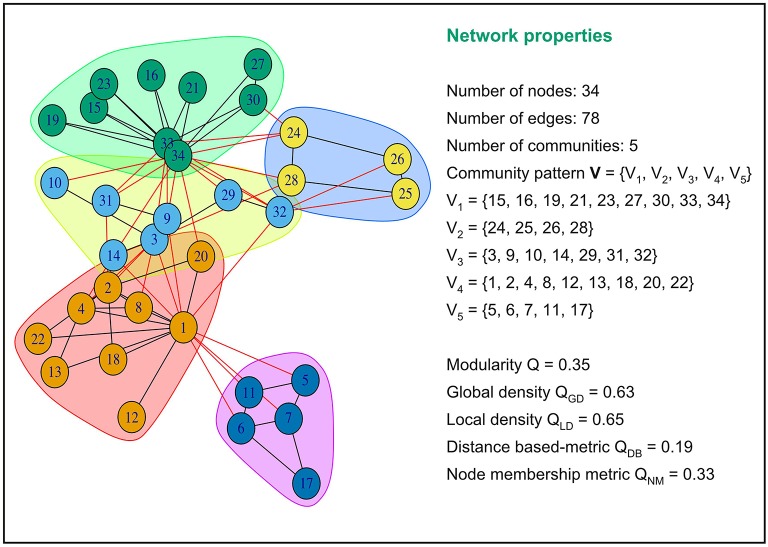
Examples of values of modularity, global density, local density, distance based and node membership metric for a specific community partition of the popular Zachary Karate's Club network.This network partition is composed of five communities or modules. Intra-community connections are colored in black and inter-community connections in red. The five community pattern measures yielded different values for this graph partition.

Brain's functional connectivity networks are known to be fundamentally modular. The neuronal regions within a community cluster have strong interconnections among themselves and weak interdependencies with neuronal regions outside the cluster. Modularity, global density, local density, distance-based, and node membership metrics try to quantify the quality of assignment of regional nodes into cohesive subgroups or neural functions. All these metrics take values between 0 and 1. A low value of the distance-based metric and a high value of the four other metrics indicate that connections between regions within community clusters are dense, and connections between regions in different community clusters are sparse. An advantage of these five community pattern metrics is that they can all be computed based solely on the connectivity of the graph. Figure 1 provides an illustration of how these metrics are computed for a particular community partitioning of the popular Zachary Karate's club network(Zachary, 1977).

Several prior studies have investigated the modular structure of resting-state structural and functional connectivity networks derived from MRI in autistic patients compared to healthy individuals. For instance, Rudie and coauthors used the Louvain algorithm(Blondel et al., 2008) to partition the brain into functional subsystems(Rudie et al., 2013). They performed additional analyses with small-world metrics, including the clustering coefficient, the characteristic path length, and modularity, to discover that children and adolescents with autism display reduction in network modularity. In another differential study, the authors used the Louvain method to partition functional brain networks into various subnetworks, and the Scale Inclusivity metric to estimate the within and between group similarity of community structures. Their main finding was that ASD is characterized with atypical connectivity in the ventro-temporal-limbic subnetworks that may underlie social impairments in ASD(Glerean et al., 2016). In a similar study, Keown et al. (2017) showed that functional subnetworks are globally atypical in ASD, together with reduced network integration and increased dispersion. Altogether, these findings suggest an aberrant reorganization of community structure in ASD, globally characterized by a reduction in modularity in persons having autism. These pioneering results provide an important indication that community patterns might be good neuromarkers for discriminating between ASD patients and healthy controls. In this study, we verified the hypothesis that the values of *Q, Q*_*GD*_, *Q*_*LD*_, *Q*_*DB*_, and *Q*_*NM*_ are significantly altered under ASD, 0,0,1which would indicate greater evidence of an altered community organization. In addition, we tested the hypothesis that these metrics can be used as features for predicting the clinical class of a particular participant.

The mathematical formulation of each of these metrics combines both the ideas of both functional integration and segregation, and they are used in this study to capture and reflect the imbalance between intra- and inter-cluster connections in autism. Using these five metrics together provides different indicators that map the brain's functional community patterns and helps highlight significant changes between health and disease states that can be leveraged by machine learning classifiers.

#### 2.2.6. Comparing Community Patterns

In this study, we used the Rand index for comparing pairs of community patterns(Rand, 1971; Steinhaeuser and Chawla, 2010). The Rand index is a statistical metric based on the community assignment of each pair of nodes and measures the degree of agreement between two community patterns **U** and **R**; it is computed using the following parameters:

- *a*: the number of pairs of nodes assigned to the same community according to both **U** and **R**
- *b*: the number of pairs of nodes assigned to the same community according to **U** but different communities according to **R**
- *c*: the number of pairs of nodes assigned to the same community according to **R** but different communities according to **U**
- *d*: the number of pairs of nodes placed in different communities according to both **U** and **R**

The sum *a* + *d* is the number of agreements between the two community patterns, whereas *b* + *c* is the number of disagreements. The Rand index between **U** and **R** is defined as

(6) $$Rand(U,R)=\frac{a+d}{\left(\begin{array}{c} N\\ 2 \end{array}\right)}.$$

### 2.3. Preprocessing Parameters

The rsfMRI data listed in Table 1 were preprocessed in the conventional order to facilitate comparison across the six datasets (Waheed et al., 2016). The data were preprocessed using the following software tools: MRIcron, SPM12, DPABI V2.3170105(Yan et al., 2016), and DPARSFA V4.3170105(Yan and Zang, 2010). The first 10 volumes of each series were discarded for signal equilibrium. Slice timing was performed to correct images for the acquisition time delay between slices of each volume, followed by head motion correction by using a six-parameter (rigid body) spatial transformation. Next, the images were normalized to the Montreal Neurological Institute EPI template and resampled into 3-mm isotropic voxels. The resulting signals were successively smoothed using a 4 mm FWHM Gaussian kernel, detrended, and band-pass filtered by using the frequency interval of 0.027–0.073 Hz (this interval was reported to be more reliable when the global signal is not regressedLiang et al., 2012). The normalized images were finally mapped with the Automated Anatomical Labeling atlas (AAL) to obtain 90 ROIs representing functional network nodes(Tzourio-Mazoyer et al., 2002). After preprocessing each dataset separately, we merged the time-series extracted from each site to form a multisite cohort.

### 2.4. Group-Level Analysis of Community Structures

To analyse group-level community patterns, first, we computed the correlation matrix for each participant from time-series data, by taking the average Pearson's correlation between all pairs of ROIs for each dataset. Next, we constructed the average correlation matrix (average brain network) for each diagnostic group. Third, these average networks were binarized using different threshold values ranging from 0.1 to 0.9. Finally, community detection was performed for each threshold value and compared between the two diagnostic groups.

Generating graphs at different sparsity levels has the advantage of allowing comparison between different graph representations at different levels of correlation. Community structures were detected using Newman's spectral modularity algorithm in the Matlab Community Detection Toolbox and visualized with BrainNet Viewer(Xia et al., 2013), a specialized Matlab toolbox for visualizing brain data. Many algorithms for community detection have been proposed, among which Newman's spectral modularity(Newman, 2006) and Infomap(Rossval and Bergstrom, 2008) have been extensively used in neuroscience studies. In this study, we used Newman's community detection algorithm because it rapidly optimizes the quality function (modularity) even with poor hardware performance, and is accurate. Community detection and evaluation were performed using the Community Detection Toolbox (ComDet)(Mitalidis et al., 2014) in Matlab. Visual inspection of networks across the datasets at different sparsities allowed the identification of general tendencies of group-level networks toward under- or overconnectivity.

### 2.5. Subject-Level Analysis

Community detection was also performed at the subject level and generated four sets of community patterns for sparsity thresholds ranging from 0.1 to 0.9. Community pattern metrics were computed for all participants in each site separately, and multisite data were generated by merging community pattern metrics computed for each site at each level of sparsity. We used a two-sample Kolmogorov-Smirnov test to assess the difference in the distribution of community quality metrics between the two diagnostic groups. This test was run on each individual dataset and on the multisite data independently. In addition, kernel density estimation (KDE) curves were plotted at each sparsity level to visualize the differences in community pattern metrics between the two clinical groups(Ledl, 2004). Additionally, a pairwise correlation analysis was performed to visualize the distribution of data from patients with ASD and controls for each value of the binarization threshold.

The differences in community partition quality indexes, although important, do not indicate how community composition or node assignments differ between the two diagnostic groups. To this end, the Rand index between each pair of individuals was computed within each clinical group according to Equation (6). The Rand index was also extended to test for group differences in each dataset and in the multisite data. Intuitively, in case of a significant group difference, the mean within-group pairwise similarity should be higher than the mean between-group pairwise similarity. Because this cannot be tested directly, a non-parametric test comparing the average within-group Rand index in the original data with that in permuted data with randomized group membership was performed. *P*-values were computed based on the number of times the within-group Rand index on the permuted data was greater than that on the original data, divided by the total number of permutations (*n* = 50,000).

To locate the brain regions that could be responsible for the difference in the Rand index between the two clinical groups, we performed another statistical test proposed by Alexander-Bloch et al. (2012). This test was implemented only on multisite data. For each network node X, the other 89 nodes were relabeled to indicate whether they are in the same module as X. These labels were subsequently compared across participants. In terms of node X's functional community, the similarity of two participants was quantified using the Rand index. Similar to the previous test, the pairwise similarity metric was used to test for nodal group difference through a permutation of group labels. The true within-group mean Rand index was computed for all within-group subject-by-subject ROI pairs. Subsequently, the labels were shuffled 10,000 times and the average permuted within-group Rand index was computed and compared with that of the real data to generate a *p*-value. Thus, for each binarization threshold, a set of 90 p-values was generated to indicate whether each ROI's community assignment was more similar across participants in the same original group than across those in randomly permuted groups.

### 2.6. Automatic Prediction of a Participant's Class

The spatial distribution of data as visualized in the subject-level analysis prompted us to verify whether the five community features (modularity, global density, local density, distance-based, and node membership) could serve as reliable predictors of ASD. Therefore, the following classification algorithms were implemented using Scikit-Learn in a Python environment: logistic regression(LR), linear discriminant analysis(LDA), k-nearest neighbors(KNN), classification and regression trees(CART), naive Bayes(NB), and support vector machines(SVM).

Given that LDA which yielded the best ASD classification accuracy with community quality metrics as features is rather often used as a supervised feature extraction method, we briefly recall the classification process using LDA. LDA classifier is derived from a probabilistic model which models, for each class or diagnostic group *k*, the class conditional distribution of the data *P*(*D*|*y* = *k*). Predictions can then be obtained by applying Bayes' rule:

(7) $$P(y=k|D)=\frac{P(D|y=k)P(y=k)}{P(D)}=\frac{P(D|y=k)P(y=k)}{\underset{l\in \{0,1\}}{\Sigma }P(D|y=l)P(y=l)}$$

and we select the class *k* which maximizes this conditional probability. More specifically, *P*(*D*|*y*) is modeled as a multivariate Gaussian distribution with density:

(8) $$P(D|y=k)=\frac{1}{{\left(2\pi \right)}^{p/2}|\Sigma {|}^{1/2}}exp\left(-\frac{1}{2}{\left(D-\mu_{k}\right)}^{t}\Sigma^{-1}(D-\mu_{k})\right)$$

where *p* is the number of features, $\mu_{k}\in {\mathbb{R}}^{p}$ the class mean vector, and Σ = *cov*[*D*] the *p*×*p* covariance matrix. To use this model as a classifier, we estimate the class priors *P*(*y* = *k*), the class means μ_*k*_ and the covariance matrix Σ from the training data(Hastie et al., 2009).

To estimate the performance of each classifier, LOO-cross-validation was used to evaluate the performance of these algorithms on each dataset at each sparsity threshold and a 10-fold cross validation was applied to multisite data at each sparsity level. The performance of each of these classifiers was reported in terms of accuracy, precision, and recall.

In order to rank community quality metrics based on their ASD predictive ability, we employed recursive feature elimination (RFE) on our best classifiers(Guyon et al., 2002). RFE is performed by recursively removing predictors and building a classification model based on those predictors that remain. It uses classification accuracy to identify predictors and (combination of predictors) that contribute the most to predicting the diagnostic group. RFE algorithm outputs a score between 0 and 1 for each predictor, and the larger the score, the more important the predictor.

### 2.7. Robustness of Community Features to Methodological Variation

Because of concerns about the effect of specific preprocessing parameters, we tested the robustness of the predictive power of the five community structure metric using a different validation dataset preprocessed with several methodological perturbations. To this end, we formed a separated multisite validation dataset composed of six additional sites, totalizing in 214 participants (ASD = 97, CTR = 117). These data were downloaded from the preprocessed version of ABIDE repository(Craddock et al., 2013). Our validation cohorts included data from the following imaging centers: Carnegie Mellon University (CMU, ASD = 14, CTR = 13), Kennedy Krieger Institute (KKI, ASD = 20, CTR = 28), Oregon Health and Science University (OHSU, ASD = 12, CTR = 14), Social Brain Laboratory (SBL, ASD = 15, CTR = 15), San Diego State University (SDS, ASD = 14 , CTR = 22) and Trinity Center for Health Sciences (TRI, ASD = 22, CTR = 25). Participant demographic information is provided in Table S3 and imaging acquisition parameters are summarized in Table S4. The downloaded imaging data derivatives were previously preprocessed using the DPARSF pipeline. The preprocessing treatments included the removal of the first ten volumes, slice timing and motion correction. Nuisance variable regression was carried out using 24 motion parameters and low-frequency drifts. Imaging signals were then band-pass filtered with a frequency range of 0.01 Hz to 0.1 Hz, without global signal correction, registered to Montreal Neuroimaging Institute template using DARTEL(Ashburner, 2007), and smoothed using a 6-mm FWHM Gaussian Kernel. The mean time courses for regions of interest were extracted for each subject based on the CC200 functional atlas which comprises 200 ROIs(Craddock et al., 2012). Functional connectomes for each participant were constructed as described previously, and community pattern metrics were computed for different network sparsity levels (0.1 ≤ *T* ≤ 0.9). We retrained KNN and LDA classifiers with features extracted for each value of the binarization threshold. Just as previously, A 10-fold cross-validation scheme was employed to evaluate these additional classifiers.

## 3. Results

### 3.1. Variations in Community Patterns

#### 3.1.1. Difference in Overall Network Structure

**Figure 2:**
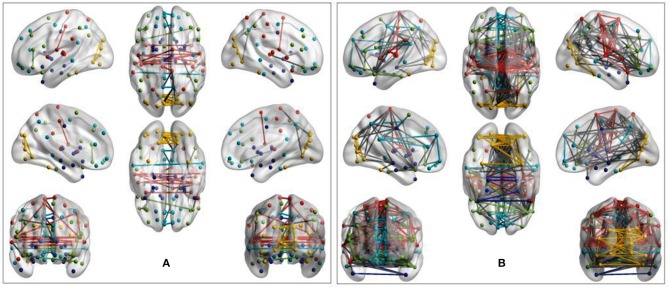
Group-average network community pattern for the LV2 dataset at sparsity threshold *T* = 0.8. ROIs are defined according to the AAL90 brain atlas and colored based on community assignments by Newman's spectral algorithm. **(A)** ASD cohort. **(B)** Control cohort. The group-level community pattern showed an overall reduction of connectivity in the brains of patients with ASD. Underconnectivity was also observed in LV1, CAL, and PIT.

**Figure 3:**
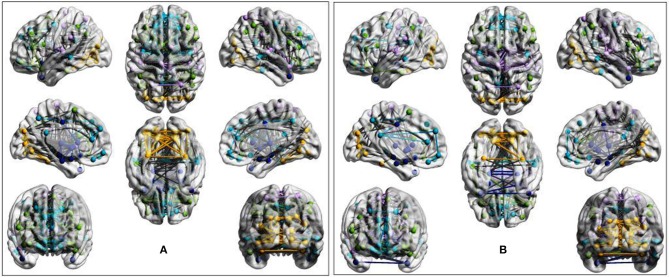
Evidence of overactivation in the community colored in yellow observed in the average STA dataset network at the sparsity threshold *T* = 0.4, despite the community pattern showing an overall preservation of network morphology. **(A)** ASD cohort. **(B)** Control cohort. ROIs are defined according to the AAL90 atlas and colored on the basis of community assignment by using the Newman's spectral algorithm. At this density level, group-average network overconnectivity was also observed in the OLI dataset.

Visual inspection of community patterns in the group-averaged networks at all sparsity levels revealed no significant difference in the number of community clusters between patients with ASD and controls. Another important observation was an overall similarity in topological cluster organization between the brains of patients with ASD and those of controls. However, at higher sparsities, over- and underactivation of some communities in the average networks of ASD cohorts was gradually observed. Notably, overall underconnectivity was found in ASD cohorts in LV1, LV2, PIT, and CAL data (Figure 2), and resting-state group network overconnectivity was observed in the OLI and STA datasets (Figure 3).

**Figure 4:**
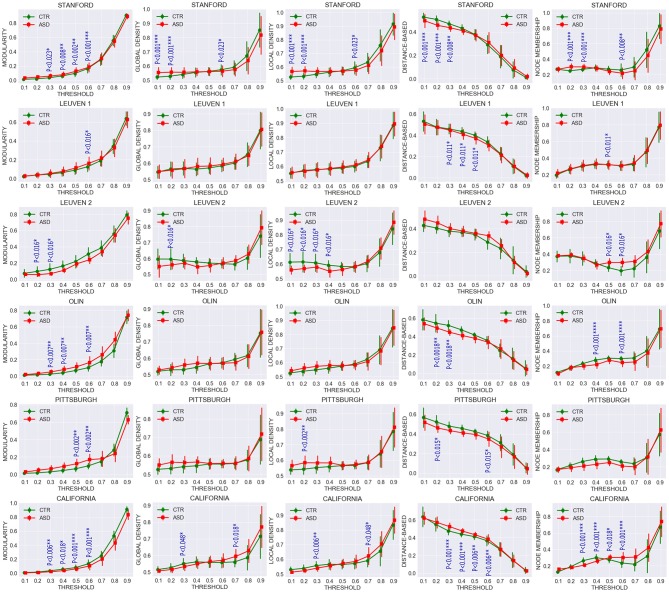
Comparing average and standard deviation of community pattern quality metrics between patients with ASD and controls for the full range of thresholds. Community quality metrics were computed for each participant, and plots were created based on the average for patients with ASD and controls. Each row represents a dataset and each column one metric. Group statistical differences were analyzed using the two-sample Kolmogorov-Smirnov test. Only significant FDR-corrected *p*-values are reported (*p* < 0.05).

To further investigate the extent to which community structures of task-free functional connectivity were altered in ASD, we computed the five descriptive community pattern metrics and generated plots of their average and standard deviation in patients with ASD and controls (Figure 4). *P*-values for mean group differences were estimated using the two-sample Kolmogorov-Smirnov test. The *p*-values obtained were subsequently FDR-corrected for multiple comparisons. Our five metrics are different ways of capturing the intuition that nodes within the same cluster should be more densely connected with each other than the rest of the network; however, they vary in their mathematical formulations. Communities were isolated through modularity maximization, and modularity was used in addition to the other four metrics to compare the resulting community patterns. Figure 4 shows that the mean difference between patients with ASD and controls is significant at several sparsities. For example, modularity is significantly higher for the ASD class in STA and CAL, whereas it remains significantly lower in OLI. The spread of the metrics around their averages also differs between the two clinical classes. Compared with the control group, in the ASD class we observed a greater spread of the values of the metrics in STA, LV1, and LV2,and a smaller one in OLI, PIT, and CAL, possibly reflecting subtypes of ASD.

**Figure 5:**
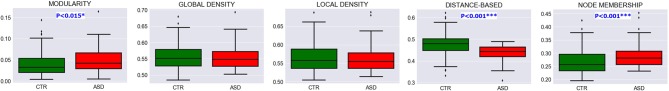
Evidence of community pattern alteration in ASD. Box plots reveal group differences in terms of community quality indexes at *T* = 0.5 in pooled data across experimental sites . *P*-values were generated with the two-sample Kolmogorov-Smirnov test and subsequently FDR-corrected.

Multisite data at *T* = 0.5 exhibited significant differences in modularity, distance-based and node membership metrics, with an overall increase in modularity and node membership, and a decrease in the three other metrics for the ASD group (Figure 5). This increase in modularity suggests that there are relatively fewer connections between clusters and more connections within clusters in patients with ASD. However, the relationship between community quality metrics and under- and overconnectivity remains unclear because a decrease in modularity was associated with underconnectivity in CAL, but with overconnectivity in STA.

#### 3.1.2. Differences in Community Composition

While the community pattern quality metrics revealed differences in the structure of resting-state functional networks, we still needed to quantify the degree of similarity of node assignment to clusters within each clinical group. To this end, Rand index similarity was computed between the ASD and control groups in the datasets (Table 2). The Rand index showed a high level of agreement and further confirmed the overall visual similarity of network structures observed (across binarization thresholds: mean Rand index = 0.82, standard deviation = 0.14). However, the Rand index permutation testing on individual subject network partitions revealed that, for some levels of sparsity, the within-group similarity of community structures of pairs of participants in the same diagnostic group is higher than would be expected if the group difference was not significant (Table 3). Moreover, this difference was also significant for multisite data (*p* = 0.033).

**Table 2:** Rand Index values measuring the degree of agreement of community structures between control and ASD groups in real data.

**T**	**STA**	**LV1**	**LV2**	**OLI**	**PIT**	**CAL**
0.1	0.69	0.64	0.76	0.84	0.97	1
0.2	0.62	0.53	0.69	0.71	0.68	0.89
0.3	0.55	0.68	0.6	0.83	0.65	0.65
0.4	0.77	0.77	0.74	0.71	0.75	0.56
0.5	0.89	0.74	0.72	0.82	0.83	0.77
0.6	0.92	0.86	0.84	0.85	0.87	0.76
0.7	0.99	0.95	0.89	0.95	0.92	0.9
0.8	1	1	0.95	0.99	0.99	0.97
0.9	1	1	0.95	1	1	1
$\bar{x}$	0.82	0.8	0.79	0.86	0.85	0.83
σ	0.17	0.17	0.12	0.11	0.13	0.16

*T, sparsity threshold; $\bar{x}$, mean; σ, standard deviation*.

**Table 3:** Rand index permutation testing revealed significant differences between ASD and CTR network community structures.

**Dataset**	**Mean within-CTR**	**Mean within-ASD**	**Mean of all within-group pairings in real data**	**Mean of all Within-group pairings with permuted labels**	***P*-value real > permuted data**
STA (*T* = 0.4)	0.557	0.548	0.552	0.547	0.017
LV1 (*T* = 0.3)	0.549	0.551	0.550	0.540	0.032
LV2 (*T* = 0.3)	0.562	0.536	0.549	0.544	0.019
OLI (*T* = 0.4)	0.555	0.558	0.557	0.551	0.046
PIT (*T* = 0.4)	0.541	0.550	0.546	0.541	0.027
CAL (*T* = 0.4)	0.569	0.564	0.565	0.558	0.001
Multisite (*T* = 0.5)	0.565	0.557	0.563	0.557	0.033

*P-values for mean group differences were estimated using a permutation test with n = 50,000 permutations*.

#### 3.1.3. Investigating Group Differences by Using Subject-Level Analysis

**Figure 6:**
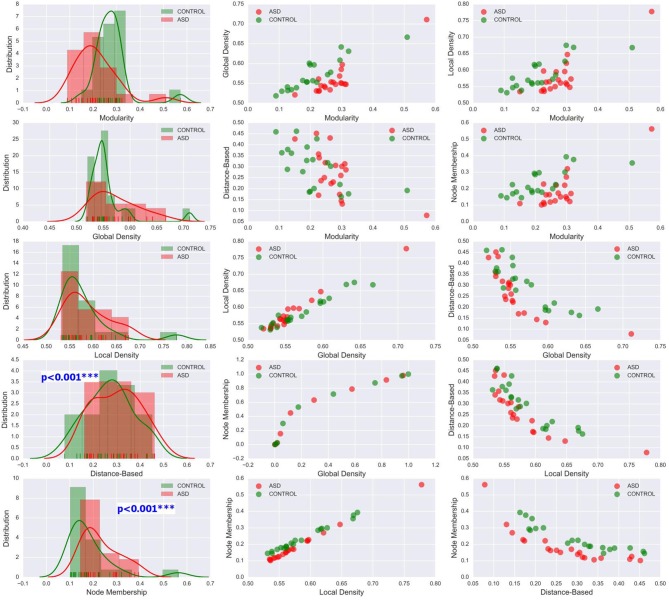
Left column: KDE plots of variations in the five community pattern metrics across subjects and clinical groups in the CAL dataset at threshold *T* = 0.4 with a Gaussian kernel bandwidth of 0.02. These plots show significant differences in the distribution of community structure metrics between the two groups. Middle and right column: organization of ASD and control group data visualized by scatter plots of all pairs of community pattern metrics.

**Figure 7:**
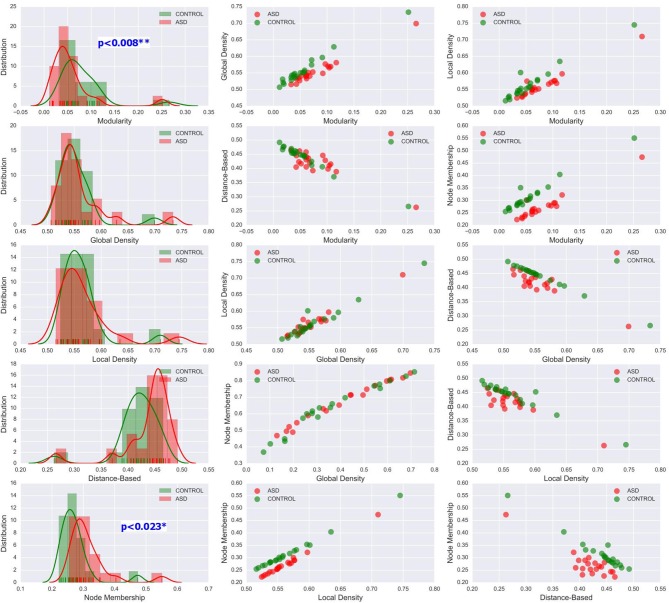
Left column: KDE plots of variations in the five community pattern metrics across subjects and clinical groups in the STA dataset at threshold *T* = 0.4 with a Gaussian kernel bandwidth of 0.02. These plots show significant differences in the distribution of community structure metrics between the two groups. Middle and right column: organization of ASD and control group data visualized by scatter plots of all pairs of community pattern metrics.

The methods used in the group-level analysis enabled qualitative and quantitative characterization of the difference between the two clinical groups. However, they do not allow the estimation of the degree of variability of community structural metrics within a clinical group compared with that across groups. Visualizing the inter-subject variability inside and across the two groups was possible using KDE plots combined with scatter plots displaying the organization of data from both clinical groups with respect to each pair of features (see Figures 6, 7 and Figures S1–S4). Although group-level analyses revealed similar patterns in the ASD and control groups, KDE showed important perturbations in the distribution of community quality metrics in all datasets and across sparsity densities. Moreover, the spatial organization displayed via feature pairing plots revealed an interesting tendency of the data from members of each of the two groups to cluster together. These two-dimensional visualizations provided an encouraging basis for applying machine learning algorithms to predict the class of a particular participant by using community structure metrics as features.

**Figure 8:**
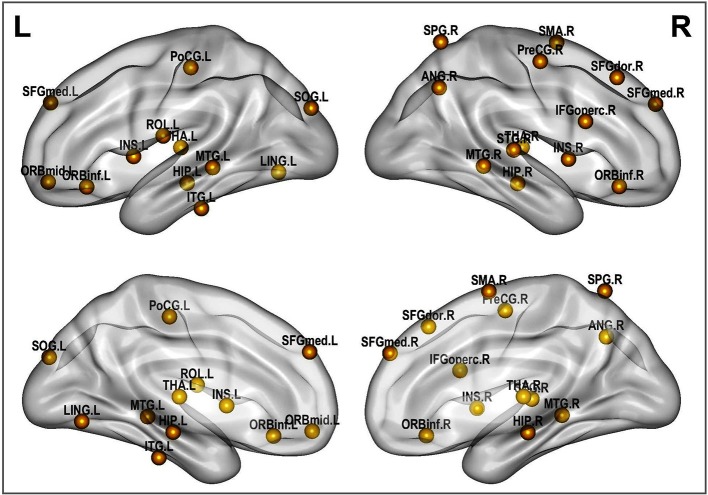
Altered brain regions in autism as revealed by ROI community assignment test. This test was conducted on pooled data across experimental sites.

A rigorous regional permutation test of community assignments adapted from Alexander-Bloch et al. (2012) was applied to multisite data and found several regions with functional community structure assignments differing significantly between the two clinical populations (see Table 4 and Figure 8). There was variability across groups in the community assignment of ROIs across all network sparsity levels. Full details of the test results are presented in Table S2.

**Table 4:** Regions displaying high disagreement between ASD and control group for community cluster assignment.

**Label**	**Region**	**Hemi**	**Coordinates**		
			**x**	**y**	**z**
2	Precental gyrus	R	41.37	−8.21	52.09
4	Superior frontal gyrus, dorsolateral	R	21.9	31.12	43.82
9	Middle frontal gyrus, orbital part	L	−30.65	50.43	−9.62
12	Inferior frontal gyrus, opercular part	R	50.2	14.98	21.41
15	Inferior frontal gyrus, orbital part	L	−35.98	30.71	−12.11
16	Inferior frontal gyrus, orbital part	R	41.22	32.23	−11.91
17	Rolandic operculum	L	−47.16	−8.48	13.95
20	Supplementary motor area	R	8.62	0.17	61.85
23	Superior frontal gyrus, medial	L	−4.8	49.17	30.89
24	Superior frontal gyrus, medial	R	9.1	50.84	30.22
29	Insula	L	−35.13	6.65	3.44
30	Insula	R	39.02	6.25	2.08
37	Hippocampus	L	−25.03	−20.74	−10.13
38	Hippocampus	R	29.23	−19.78	−10.33
47	Lingual gyrus	L	−14.62	−67.56	−4.63
49	Superior occipital gyrus	L	−16.54	−84.26	28.17
57	Postcentral gyrus	L	−42.46	−22.63	48.92
60	Superior parietal gyrus	R	26.11	−59.18	62.06
66	Angular gyrus	R	45.51	−59.98	38.63
77	Thalamus	L	−10.85	−17.56	7.98
78	Thalamus	R	13	−17.55	8.09
82	Superior temporal gyrus	R	58.15	−21.78	6.8
85	Middle temporal gyrus	L	−55.52	−33.8	−2.2
86	Middle temporal gyrus	R	57.47	−37.23	−1.47
89	Inferior temporal gyrus	L	−49.77	−28.05	−23.17

*This test was performed on merged data across experimental sites. Hemi, hemisphere; L, left; R, right. These regions can be visualized in Figure 8*.

### 3.2. Single Subject Clinical Group Prediction

As previously mentioned in this document, six classification algorithms were implemented by using Scikit-learn in a Python environment to investigate whether the community structure quality metrics of the participant's resting-state functional connectivity networks alone could predict the clinical group of a particular participant. Among the classification algorithms, LDA and KNN yielded the best results with the LOO-cross-validation test; the performances of these two algorithms are reported in Table 5. LDA achieved peak accuracy ranging from 74.86% (CAL) to 85.16% (STA). KNN obtained a range of peak accuracy from 68.42% (PIT) to 76.12% (STA). However, these results were obtained at different network sparsity levels. We merged all the five community pattern features computed for each sparsity level, retrained the classifiers and performed a 10-fold cross-validation test. Multisite data yielded peak accuracy at *T* = 0.5 (65.66% for KNN and 74.86% for LDA). Compared with recent autism classification studies, this study obtained a relatively high classification accuracy with the lowest number of predictors (Table 6).

**Table 5:** Classification performance on our data cohorts by using the five community pattern descriptors as features with KNN and LDA algorithms.

**Algorithm**	**KNN**			**LDA**		
**Dataset**	**Accuracy**	**Precision**	**Recall**	**Accuracy**	**Precision**	**Recall**
STA (*T*=0.2)	76.12	74.48	72.91	85.16	84.25	83.95
LV1 (*T* = 0.3)	70.31	66.83	61.00	82.77	80.10	81.89
LV2 (*T* = 0.3)	69.69	48.17	51.67	81.33	80.79	80.29
OLI (*T* = 0.4)	74.44	77.58	72.01	80.28	79.08	80.04
PIT (*T* = 0.3)	68.42	57.50	52.49	79.59	78.03	78.77
CAL (*T* =0.6)	72.00	73.33	71.21	83.35	82.92	83.01
Multisite(*T* = 0.5)	65.66	59.00	59.00	74.86	76.07	71.67

*T, Threshold; KNN, K-nearest neighbor; LDA, linear discriminant analysis. We only report the sparsity thresholds that yielded the highest classification accuracy*.

**Table 6:** Comparing our classification results with recent works.

**Types of features**	**# of features**	**Classifier**	**# of subjects (ASD, CTR, Total)**	**Peak accuracy %**	**References**
Functional connectivity	26,393,745	Thresholding	(40, 40, 80)	79.0	Anderson et al., 2011
ICA components	10	Linear Regression	(20, 20, 40)	78.0	Uddin et al., 2011
Functional connectivity among 7266 ROIs	26,400,000	General Linear Model	(447, 517, 964)	60	Nielsen et al., 2013
Functional connectivity among 220 ROIs	24,090	Random Forest	(126, 126, 252)	91	Chen et al., 2015
Functional connectivity among 90 ROIs	4005	Probabilistic Neural Network	(312, 328, 640)	90	Iidaka, 2015
Functional connectivity	Variable	Support Vector Machine	(59, 89, 148)	76.7	Plitt et al., 2015
Functional connectivity among 84 ROIs	7,056	Support Vector Classification	(468, 403, 871)	67	Abraham et al., 2017
Functional connectivity among ROIs	600	Deep neural network	(505, 530, 1035)	70	Heinsfeld et al., 2018
HOG and personal characteristic data	47	Support Vector Machine	(538, 573, 1111)	65	Ghiassian et al., 2016
ROIs HMMs likelihoods	114	SVM	(121, 171, 292)	75.86	Jun et al., 2019
Time series	90	LSTM	(529, 571, 1100)	68.5	Dvornek et al., 2017
Community metrics from 90-ROI networks	5	Linear Discriminant Analysis	(117, 118, 235)	74.86	This work

*HOG, Histogram of Oriented Gradients; LSTM, Long Short-Term Memory network; HMM, Hidden Markov Models*.

### 3.3. Feature Importance

To determine which community pattern features were most predictive, we performed Recursive Feature Elimination (RFE) on in-site as well as multisite data with stratified-10-fold cross-validation. This procedure used our LDA model to rank the five community pattern metrics according to their predictive performance during the classification process. For CAL, LV1, LV2, and PIT, the order of feature importance are global density, node membership, local density, modularity and distance-based metric, starting from the most important feature. For OLI and STA, important features are global density, node membership, local density, distance-based metric, modularity and node memebership. RFE on multisite data showed that local density was the most important predictor (score = 0.95), followed by global density (score = 0.75) and node membership (score = 0.5). Modularity and distance-based metric were the less predictive features with a score of 0.25 and 0.10, respectively.

### 3.4. Robustness to Methodological Variation

Finally, we examined how community pattern metrics would perform on novel datasets and under a different set of preprocessing parameters, including the head motion correction parameter, the smoothing parameter, the bandpass filtering frequency range and the ROI parcellation atlas. Our validation cohorts were used for this purpose. Group-level analyses of community structure for validation datasets are summarized in Tables S5, S6. Subject-level analyses of community quality metrics are recapitulated in Figures S5–S11. Single validation sites obtained peak classification accuracy of 68.12% for CMU (*T* = 0.3), 76.23% for KKI (*T* = 0.6), 82.02% for OHSU (*T* = 0.4), 71.09% for SBL (*T* = 0.7), 80.73% for SDSU (*T* = 0.3) and 72.58% for TRINITY (*T* = 0.8), using LDA and leave-one-out cross-validation method. Again, classification accuracies obtained on in-site data using KKN were consistently lower than those obtained with LDA. For multisite classification on the whole validation set, the highest classification obtained is 75.04% (*T* = 0.4) obtained with LDA and 10-fold cross-validation (see Table 7 for full classification results on the whole validation dataset). Taken together, these results suggest that the discriminative capability of community patterns metrics used in this study is relatively well-preserved on novel datasets and under alternative preprocessing choices. However, the range of filtering thresholds values that yielded peak classification accuracy differs considerably between experimental and validation data. Furthermore, the most important features differs sightly from those obtained with experimental data. RFE applied on the whole validation dataset revealed global density was most discriminative (score = 0.90), followed by local density, node membership, modularity and distance-based metric that obtained predictive scores of 0.80, 0.70, 0.30, and 0.08, respectively.

**Table 7:** Classification performance on the entire validation set by using the five community pattern descriptors as features with KNN and LDA algorithms.

**Threshold**	**KNN**			**LDA**		
	**Accuracy**	**Precision**	**Recall**	**Accuracy**	**Precision**	**Recall**
0.1	58.25	59.23	59.27	65.14	64.34	64.89
0.2	55.69	56.61	61.55	68.85	68.15	68.18
0.3	65.95	64.29	64.07	69	70.15	71.58
0.4	65.97	66.13	64.13	75.04	73.16	74.28
0.5	66.16	67.81	64.51	72.49	71.44	72.77
0.6	64.33	62.45	63.18	71.16	70.98	69.14
0.7	64.17	65.3	64.41	67.24	66.88	65.69
0.8	58.09	54.75	56.85	65.55	65.11	63.13
0.9	55.75	55.78	54.07	66.82	67.12	66.33

*KNN, K-nearest neighbor; LDA, linear discriminant analysis*.

## 4. Discussion

This study addressed two separate but closely related problems: the characterization of differences in the resting-state functional network community patterns between patients with ASD and age-matched controls and the single-subject prediction of this same neurological disorder. We repeated the same analyses on six experimental datasets originating from different sites and including participants of different ages, obtained using different imaging acquisition parameters. We also applied this same analysis pipeline on a multisite cohort formed by merging experimental data from the six sites. We used five community pattern comparison metrics to reach more robust conclusions. The major findings of our investigation are as follows: (1) Underconnectivity in the networks from patients with ASD compared with controls was found in four of the six datasets (LV1, LV2, CAL, and PIT) and overconnectivity was observed in two (STA and OLI); (2) statistical analyses provided strong evidence for alterations in functional community patterns in ASD, as determined using community quality indexes; (3) group-averaged networks from patients with ASD and controls exhibited a high level of Rand index similarity; however, testing of an individual's community structures revealed significant differences in cluster composition between the two classes; (4) the differences in community assignments was driven by specific regional nodes, most of which are known to be impaired in ASD; (5) community quality metrics yielded a minimum of 79% peak classification accuracy for experimental datasets, and 76% for validation datasets. Classification accuracy was lower for multisite data (74.86% for experimental data and 75.04 for validation data). The originality of our findings stems from the use of four complex network metrics that have not been previously used to analyse the functional modular organization of the human brain using neuroimaging data. To the best of our knowledge, this study is the first to reveal that the modular organization metrics alone are used to design individual subject predictive models of neurological disorders.

Our five metrics are derived from the concept of community structures in complex networks. While the notion of community structure has not been explicitly defined, community quality metrics formalize the intuition that while nodes in a community are densely interconnected, they are only sparsely connected to the rest of the network. Many quality functions have been proposed to formalize this intuition, which may suggest that none of them is completely satisfactory. This justifies the use of five metrics in this study to investigate community patterns in ASD. Although the five metrics are formalizations of the same intuition, they vary considerably in their mathematical formulations. Modularity *Q* is the fraction of the edges that fall within the given clusters or communities minus the expected fraction if connections were distributed randomly. Global density *Q*_*GD*_ is the average of global inner density and global outer antidensity. Global inner density is the sum of all within-cluster connections over all communities, divided by the number of all possible internal edges; global outer antidensity is evaluated as one minus the number of edges between the given clusters divided by the number of all possible bridge connections. Local density *Q*_*LD*_ is the average of a cluster's (local) inner densities and its (local) outer antidensities weighted by a term proportionate to the cluster's size (to ensure that small dense clusters do not influence the total clustering quality disproportionately). *Q*_*DB*_ tries to formalize the theoretical hypothesis of perfect community structure stipulating that any two nodes within the same community are connected and any two nodes in different communities are not connected. The node membership quality function computes the average (over all nodes of the graph) of a statistic that measures the likelihood of each node to belong to his assigned cluster and not other clusters. We can see that each of these metrics summarizing whole-brain connectivity with a single statistic captures a specific aspect of the quality of functional community structures. Considering that all these five measures of functional segregation are highly sensitive to every single connection and every meaningful grouping of connections in the graph, they provide a robust method for comparing connectivity between normal and pathological individuals. Nevertheless, although both underconnectivity and overconnectivity were discovered in our datasets, any potential relationship between these two potential subtypes of autism and functional community patterns remains unclear. This diversity in findings may be explained by the multifaceted manners in which ASD manifests across individuals.

With respect to functional connectivity differences between the ASD and control groups, our results are in agreement with previous findings and support the dysconnectivity theory of autism. Early studies on functional connectivity at rest in autism tended to support the underconnectivity theory, whereas a few recent studies have reported either over connectivity or evidence for both(Hull et al., 2016). However, most of these studies have focused only on specific ROIs or resting-state networks; few have addressed connectivity differences at the whole-brain level by using community detection and analysis over multiple datasets, as was done in the present study. While statistical testing revealed significant differences in the network structure and community composition, a test at the node level indicated that this difference was caused by several brain regions. These brain regions include the insula, thalamus, hippocampus, lingual gyrus, middle temporal gyrus and other functional areas that are known to be impaired in autism(Nielsen et al., 2013; Chen et al., 2016; Wang et al., 2017; Heinsfeld et al., 2018).

As shown in Table 6, descriptive community pattern metrics yielded over 79% accuracy on all of the individual datasets. Moreover, they yielded a maximum accuracy of 75.04% on a different multisite validation dataset (Table 7), thus proving to be robust, viable predictors of autism. While comparing accuracies across studies is not always straightforward, depending as they do on additional parameters such as the number of participants recorded and the preprocessing pipeline used, there is evidence that our classification significantly outperforms, even at the group level, recent approaches that used fine-scaled pairwise correlations on single-site data. Furthermore, our classification was achieved with the lowest number of features.

Despite the encouraging prediction performance obtained in this study, we do not advocate these metrics as potential ASD clinical biomarkers. One of their limitations for this purpose is that those network indexes are not complete invariants, in the sense that non-equivalent graph structures can yield the same values in those metrics. While this limitation is somewhat alleviated in this work by the use of several measures, they nevertheless fall short of neuromarker standards(Plitt et al., 2015). Another major limitation is their great dependence on network filtering threshold for which there is no objective selection criterion. That said, community quality patterns remain a valuable tool for investigating network connectivity disruptions in ASD pathology and anticipating the polarity of a particular participant before using the recommended diagnostic methods. Further research may provide a solid basis for their clinical application in the future. Autism spectrum encompasses several neurological disorders and manifests itself through a wide range of symptoms and different characteristics. The way the brain's architecture breaks down under the effects of autism is subtle and complex. A single metric, modularity, for example, is not enough to capture all the changes in brain structure across the spectrum. The use of several structural metrics is, therefore, more appropriate to capture and identify this disease.

Classifiers designed based on features extracted from ABIDE rsfMRI data typically perform better on single-site data than multisite data. Decreased accuracy in multisite data could be attributed to ASD subtypes or other heterogeneities across the ABIDE sites(Di Martino et al., 2014). Different studies employed different approaches for utilizing multisite data for ASD classification in the literature. One approach is to learn biomarkers of neurological status and perform separate classification at individual sites and then combine the results in a meta-analysis(Chen et al., 2016). Another approach consists of treating multisite data as a single, homogeneous dataset(Nielsen et al., 2013). These two approaches were used in this study to assess the viability of functional network community pattern metrics as predictors of ASD. While these two approaches fail to account for the variability that has been proven to be significant between sites, their use in this study provides preliminary evidence for community quality metrics as potential predictors of autism. Recent approaches for combining imaging data from multiple sites leverage similarity across sites while accounting for individual site differences through a joint optimization(Wang et al., 2017; Heinsfeld et al., 2018). While these novel approaches yield better classification accuracy in multisite studies, they may not be suited for studies that extract imaging features based on global connectivity indexes.

In this study, community detection was performed by optimizing the modularity quality function. Then the community quality indexes were calculated based on the found community structures. Given the results for feature importance, it is interesting to see that modularity is one of the least important predictive features for the ASD classifiers. One might then suspect that optimizing some of the other quality functions might lead to communities that yield better discrimination between persons with ASD and typical controls. In this work, the modularity maximization algorithm was chosen for community identification mostly because of its good performance on functional brain networks in previous studies. We cannot, therefore, rule out the fact that the predictive power of the other quality measures is a consequence of using modularity for the original clustering. Using an alternative graph clustering algorithm such as Infomap(Rossval and Bergstrom, 2008) to perform the original clustering could be useful for verifying this hypothesis. In addition, it would be interesting to conduct a comparative study where the initial community detection is performed by optimizing each of the other quality functions and then computing and using all the metrics as features for classification. This, however, is beyond the scope of this paper.

One limitation of the classification framework proposed in this study, and graph-based approaches in general, is that the classification results are very dependent on thresholding parameter *T*. Graph screening is a major and most recurring issue for the binarization of functional brain networks. In this study, we performed a systematic analysis of functional brain networks for increasing threshold values ranging from 0.1 to 0.9, as there is no objective criterion for determining an interval of thresholds for which community quality metrics would remain relatively stable. Our classification results on single and multisite data show that, broadly, threshold values falling between 0.3 and 0.6 yielded the best classification accuracies (see Tables 5, 7). This suggests that brain networks that are either too densely connected or too sparse are not good choices for reaching “optimal” classification accuracy on new data. Still, finding a general rule for choosing the best network filtering threshold remains a challenging endeavorDe Vico Fallani et al. (2014). A potential good workaround solution to the threshold problem could be to perform community detection and compute metrics directly from unfiltered networks. A drawback of this solution could be the challenge of defining and interpreting communities in the context of signed networks with positive and negative connections.

Other limitations of this study include the fact that the same spatial normalization template was used for all participants despite age differences in the experimental populations. Detection of regional distortions could probably be more accurate by using multiple brain templates adapted to different age ranges. Also, many subjects with ASD were on medication at the time of scanning, and it cannot be ruled out that treatments could influence resting-state functional connectivity community patterns in these individuals. Third, community detection was performed on unweighted networks, ignoring the potential significance of the information carried by edge weights. Finally, we used values of Pearson's correlation coefficient as node weights before binarization; however, different correlation metrics may yield different graph representations of the same datasets and yield different characterizations of functional connectivity differences in ASD. Further studies are necessary to investigate community pattern differences in ASD by using weighted network representation. Further studies are also warranted to determine the effects of different correlation metrics and other network construction techniques on resting-state functional network community patterns.

## 5. Conclusion

We propose a framework to characterize and discriminate patients with autism spectrum disorder from normal control subjects. Our approach is based on graph-based feature extraction. A combination of five well-selected community pattern quality indexes was used as features for classification. In addition, various statistical tests were applied to evaluate the overall network topology and community composition in ASD at the group as well as subject levels. Results for functional connectivity difference between autistic patients and normal subjects were consistent with existing studies, revealing both patterns of underconnectivity and overconnectivity. In particular, we demonstrated that the modular structure is significantly disturbed in patients with ASD. More importantly, we showed that the discriminative power of the modular structure as captured by the selected metrics is comparatively high, lending further credence to the dysconnectivity theory of this condition, for which network connectivity patterns are increasingly being considered as potential biomarkers.

## Author Contributions

All authors have made a substantial contribution to this work and approved it for publication. In particular, YS and TE designed the experimental framework, implemented computer code and drafted the manuscript and HL revised the manuscript.

### Conflict of Interest Statement

The authors declare that the research was conducted in the absence of any commercial or financial relationships that could be construed as a potential conflict of interest.
