# Supplementary material for: Characterizing and Predicting Autism Spectrum Disorder by Performing Resting-State Functional Network Community Pattern Analysis
Source: Front Hum Neurosci. 2019 Jun 14;13:203. doi: 10.3389/fnhum.2019.00203 (PMC6587437; doi:10.3389/fnhum.2019.00203)
Supplement: Supplementary file 1 [file Data_Sheet_1.PDF]

Supplementary Material: Characterising and predicting autism spectrum disorder by performing resting-state functional network community pattern analysis

1 SUPPLEMENTARY MATERIAL FOR EXPERIMENTAL DATASETS

1.1 Supplementary Tables

Table S1: Imaging data acquisition parameters useful for preprocessing (experimental datasets)

	STANFORD	LEUVEN 1	LEUVEN 2	OLIN	PITTSBURGH	CALTECH
Scanner	GE 3T	PHI 3T	PHI 3T	SIE MR 2004A	SIE MR A30	SIE MR B17
slice orientation	Oblique	Transversal	Transversal	Transversal	Transversal	Transversal
#slices	29	32	32	29	29	34
Voxel size (mm^3)	3.125×3.125×4.500	3.58×3.59×4.0	3.58×3.59×4.0	3.4×3.4×4.0	3.1×3.1×4.0	3.5×3.5×3.5
Resolution	64 × 64	64× 64	64 × 64	64×64	64×64	64×64
TR(ms)/TE(ms)	2000/30	1667/33	1667/33	1500/27	1500/25	2000/30
Flip angle (○)	80	90	90	60	70	75
Field of view (mm)	200	230	230	220	200	224

GE 3T = GE Signa 3T, PHI 3T = Philips Intera 3T, SIE MR 2004A = Siemens Magnetom Allegra Syngo MR 2004A, SIE MR A30 = Siemens Magnetom Allegra Syngo MR A30, SIE MR B17 = Siemens Magnetom Triotim Syngo MR B17

Table S2: ROI dissimilarity test results (multisite, experimental data)

ROI's full name	Abbreviation	T=0.1	T=0.2	T=0.3	T=0.4	T=0.5	T=0.6	T=0.7	T=0.8	T=0.9
Precental gyrus	PreCG.L	0.966	0.326	0.939	0.088	0.603	0.320	0.523	0.358	0.801
Precental gyrus	PreCG.R	0.888	0.084	0.881	0.699	0.998	0.377	0.910	0.645	**0.004**
Superior frontal gyrus, dorsolateral	SFGdor.L	0.532	0.970	0.221	0.960	0.531	0.083	0.703	0.417	0.162
Superior frontal gyrus, dorsolateral	SFGdor.R	0.053	**0.024**	0.154	0.620	0.410	0.119	0.784	0.195	0.117
Superior frontal gyrus, orbital part	ORBsup.L	0.824	0.992	0.612	0.631	0.296	0.272	0.175	0.584	0.834
Superior frontal gyrus, orbital part	ORBsup.R	0.102	0.314	0.984	0.280	0.776	0.424	0.056	0.215	0.886
Middle frontal gyrus	MFG.L	0.452	0.540	0.797	0.973	0.102	0.681	0.944	0.326	0.118
Middle frontal gyrus	MFG.R	0.627	0.687	0.679	0.446	0.399	0.837	0.790	0.208	0.787
Middle frontal gyrus, orbital part	ORBmid.L	0.326	0.013	0.870	0.994	0.411	0.775	0.605	0.982	0.167
Middle frontal gyrus, orbital part	ORBmid.R	0.940	0.106	0.628	0.555	0.709	0.360	0.858	0.848	0.164
Inferior frontal gyrus, opercular part	IFGoperc.L	0.983	0.548	0.166	0.853	0.672	0.959	0.439	0.323	0.947
Inferior frontal gyrus, opercular part	IFGoperc.R	0.655	0.919	0.415	0.062	0.165	0.029	0.964	0.477	0.590
Inferior frontal gyrus, triangular part	IFGtriang.L	0.210	0.661	0.598	0.121	0.301	0.067	0.514	0.091	0.756
Inferior frontal gyrus, triangular part	IFGtriang.R	0.712	0.876	0.185	0.560	0.932	0.127	0.227	0.468	0.812
Inferior frontal gyrus, orbital part	ORBinf.L	0.852	0.007	0.422	0.592	0.225	0.201	0.518	0.498	0.596
Inferior frontal gyrus, orbital part	ORBinf.R	0.386	0.701	0.931	0.018	0.221	0.173	0.350	0.834	0.741
Rolandic operculum	ROL.L	0.556	0.387	0.218	0.059	0.369	0.040	0.921	0.979	0.515
Rolandic operculum	ROL.R	0.438	0.188	0.817	0.056	0.076	0.509	0.235	0.660	0.189
Supplementary motor area	SMA.L	0.261	0.847	0.436	0.677	0.565	0.286	0.859	0.730	0.623
Supplementary motor area	SMA.R	0.177	0.440	0.849	0.237	0.693	0.364	0.773	0.563	0.026
Olfactory cortex	OLF.L	0.864	0.402	0.637	0.762	0.182	0.638	0.776	0.120	0.617
Olfactory cortex	OLF.R	0.934	0.306	0.917	0.396	0.238	0.262	0.746	0.117	0.417
Superior frontal gyrus, medial	SFGmed.L	0.901	0.266	0.822	0.587	0.350	0.747	0.014	0.646	0.037
Superior frontal gyrus, medial	SFGmed.R	0.040	0.479	0.525	0.513	0.289	0.071	0.172	0.152	0.504
Superior frontal gyrus, medial orbital	ORBsupmed.L	0.978	0.420	0.649	0.070	0.059	0.247	0.977	0.127	0.476
Superior frontal gyrus, medial orbital	ORBsupmed.R	0.747	0.976	0.248	0.533	0.786	0.300	0.438	0.251	0.572
Gyrus rectus	REC.L	0.841	0.728	0.164	0.701	0.090	0.796	0.889	0.501	0.601

Gyrus rectus	REC.R	0.345	0.424	0.546	0.915	0.322	0.584	0.858	0.388	0.453
Insula	INS.L	0.657	0.019	0.011	0.006	0.695	0.994	0.432	0.080	0.628
Insula	INS.R	0.533	0.023	0.048	0.682	0.171	0.420	0.538	0.368	0.604
Anterior cingulate and paracingulate gyri	ACG.L	0.427	0.709	0.261	0.499	0.321	0.958	0.265	0.762	0.181
Anterior cingulate and paracingulate gyri	ACG.R	0.689	0.993	0.683	0.240	0.105	0.927	0.313	0.527	0.420
Median cingulate and paracingulate gyri	DCG.L	0.615	0.359	0.286	0.716	0.671	0.777	0.305	0.854	0.164
Median cingulate and paracingulate gyri	DCG.R	0.865	0.506	0.821	0.109	0.811	0.255	0.169	0.757	0.258
Posterior cingulate gyrus	PCG.L	0.072	0.398	0.492	0.237	0.197	0.817	0.744	0.193	0.295
Posterior cingulate gyrus	PCG.R	0.542	0.903	0.484	0.878	0.213	0.613	0.430	0.082	0.294
Hippocampus	HIP.L	0.930	0.017	0.788	0.770	0.011	0.393	0.455	0.094	0.025
Hippocampus	HIP.R	0.222	0.474	0.403	0.380	0.237	0.044	0.286	0.615	0.033
Parahippocampal gyrus	PHG.L	0.574	0.513	0.777	0.714	0.736	0.509	0.667	0.316	0.838
Parahippocampal gyrus	PHG.R	0.480	0.808	0.255	0.088	0.920	0.348	0.545	0.975	0.816
Amygdala	AMYG.L	0.434	0.361	0.558	0.306	0.893	0.174	0.638	0.817	0.231
Amygdala	AMYG.R	0.476	0.343	0.413	0.667	0.576	0.926	0.808	0.661	0.457
Calcarine fissure and surrounding cortex	CAL.L	0.181	0.850	0.994	0.483	0.673	0.796	0.519	0.163	0.384
Calcarine fissure and surrounding cortex	CAL.R	0.979	0.982	0.791	0.369	0.376	0.943	0.572	0.755	0.547
Cuneus	CUN.L	0.279	0.921	0.852	0.970	0.626	0.835	0.415	0.880	0.488
Cuneus	CUN.R	0.348	0.154	0.777	0.936	0.887	0.378	0.507	0.428	0.679
Lingual gyrus	LING.L	0.960	0.306	0.048	0.884	0.937	0.574	0.430	0.818	0.022
Lingual gyrus	LING.R	0.883	0.261	0.305	0.768	0.702	0.622	0.711	0.551	0.381
Superior occipital gyrus	SOG.L	0.086	0.366	0.184	0.753	0.088	0.015	0.095	0.188	0.500
Superior occipital gyrus	SOG.R	0.577	0.166	0.658	0.184	0.326	0.375	0.204	0.287	0.538
Middle occipital gyrus	MOG.L	0.321	0.220	0.259	0.447	0.659	0.164	0.404	0.179	0.299
Middle occipital gyrus	MOG.R	0.737	0.485	0.332	0.184	0.284	0.686	0.760	0.239	0.545
Inferior occipital gyrus	IOG.L	0.375	0.421	0.450	0.169	0.073	0.773	0.683	0.553	0.715
Inferior occipital gyrus	IOG.R	0.524	0.815	0.135	0.947	0.464	0.200	0.743	0.458	0.700
Fusiform gyrus	FFG.L	0.500	0.867	0.876	0.166	0.076	0.630	0.145	0.275	0.448

Fusiform gyrus	FFG.R	0.236	0.204	0.221	0.313	0.692	0.378	0.085	0.219	0.926
Postcentral gyrus	PoCG.L	0.042	0.097	0.795	0.333	0.689	0.295	0.147	0.961	0.074
Postcentral gyrus	PoCG.R	0.658	0.166	0.880	0.061	0.499	0.628	0.680	0.967	0.273
Superior parietal gyrus	SPG.L	0.898	0.905	0.132	0.376	0.844	0.714	0.371	0.098	0.524
Superior parietal gyrus	SPG.R	0.010	0.589	0.949	0.939	0.529	0.381	0.552	0.276	0.642
Inferior parietal, but supramarginal and angular gyr	IPL.L	0.057	0.997	0.366	0.862	0.407	0.243	0.240	0.129	0.673
Inferior parietal, but supramarginal and angular gyr	IPL.R	0.288	0.283	0.545	0.699	0.154	0.599	0.390	0.547	0.893
Supramarginal gyrus	SMG.L	0.357	0.674	0.993	0.082	0.434	0.786	0.441	0.897	0.425
Supramarginal gyrus	SMG.R	0.136	0.519	0.700	0.632	0.073	0.771	0.202	0.067	0.659
Angular gyrus	ANG.L	0.215	0.138	0.539	0.066	0.340	0.110	0.814	0.688	0.444
Angular gyrus	ANG.R	0.960	0.154	0.048	0.406	0.893	0.056	0.422	0.545	0.741
Precuneus	PCUN.L	0.107	0.947	0.271	0.557	0.601	0.428	0.317	0.764	0.174
Precuneus	PCUN.R	0.349	0.273	0.588	0.650	0.670	0.873	0.806	0.739	0.372
Paracentral lobule	PCL.L	0.154	0.527	0.173	0.156	0.614	0.713	0.527	0.195	0.317
Paracentral lobule	PCL.R	0.752	0.216	0.598	0.157	0.534	0.374	0.096	0.422	0.431
Caudate nucleus	CAU.L	0.298	0.558	0.718	0.260	0.152	0.559	0.375	0.561	0.239
Caudate nucleus	CAU.R	0.848	0.431	0.170	0.472	0.175	0.575	0.081	0.147	0.402
Lenticular nucleus, putamen	PUT.L	0.107	0.773	0.522	0.317	0.350	0.822	0.120	0.532	0.474
Lenticular nucleus, putamen	PUT.R	0.913	0.086	0.410	0.396	0.188	0.818	0.368	0.870	0.555
Lenticular nucleus, pallidum	PAL.L	0.822	0.271	0.106	0.657	0.175	0.363	0.216	0.422	0.142
Lenticular nucleus, pallidum	PAL.R	0.156	0.636	0.614	0.196	0.987	0.722	0.335	0.086	0.511
Thalamus	THA.L	0.313	0.239	0.804	0.614	0.492	0.777	0.085	0.648	0.015
Thalamus	THA.R	0.686	0.019	0.073	0.630	0.178	0.557	0.827	0.266	0.698
Heschl gyrus	HES.L	0.727	0.236	0.102	0.462	0.619	0.207	0.932	0.631	0.306
Heschl gyrus	HES.R	0.314	0.190	0.898	0.418	0.286	0.504	0.227	0.248	0.143
Superior temporal gyrus	STG.L	0.688	0.702	0.720	0.275	0.720	0.615	0.289	0.318	0.947
Superior temporal gyrus	STG.R	0.002	0.255	0.829	0.679	0.682	0.592	0.484	0.314	0.943
Temporal pole: superior temporal gyrus	TPOsup.L	0.122	0.289	0.702	0.301	0.257	0.136	0.145	0.199	0.586

Temporal pole: superior temporal gyrus	TPOsup.R	0.257	0.150	0.057	0.430	0.959	0.136	0.930	0.500	0.070
Middle temporal gyrus	MTG.L	0.379	0.908	0.088	0.205	0.480	0.480	0.981	0.017	0.021
Middle temporal gyrus	MTG.R	0.637	0.981	0.387	0.777	0.341	0.759	0.130	0.684	0.032
Temporal pole: middle temporal gyrus	TPOmid.L	0.440	0.640	0.333	0.992	0.497	0.912	0.544	0.576	0.554
Temporal pole: middle temporal gyrus	TPOmid.R	0.595	0.056	0.926	0.393	0.372	0.895	0.522	0.245	0.213
Inferior temporal gyrus	ITG.L	0.180	0.970	0.249	0.941	0.203	0.036	0.798	0.710	0.225
Inferior temporal gyrus	ITG.R	0.886	0.173	0.234	0.215	0.240	0.791	0.448	0.325	0.253

1.2 Supplementary Figures

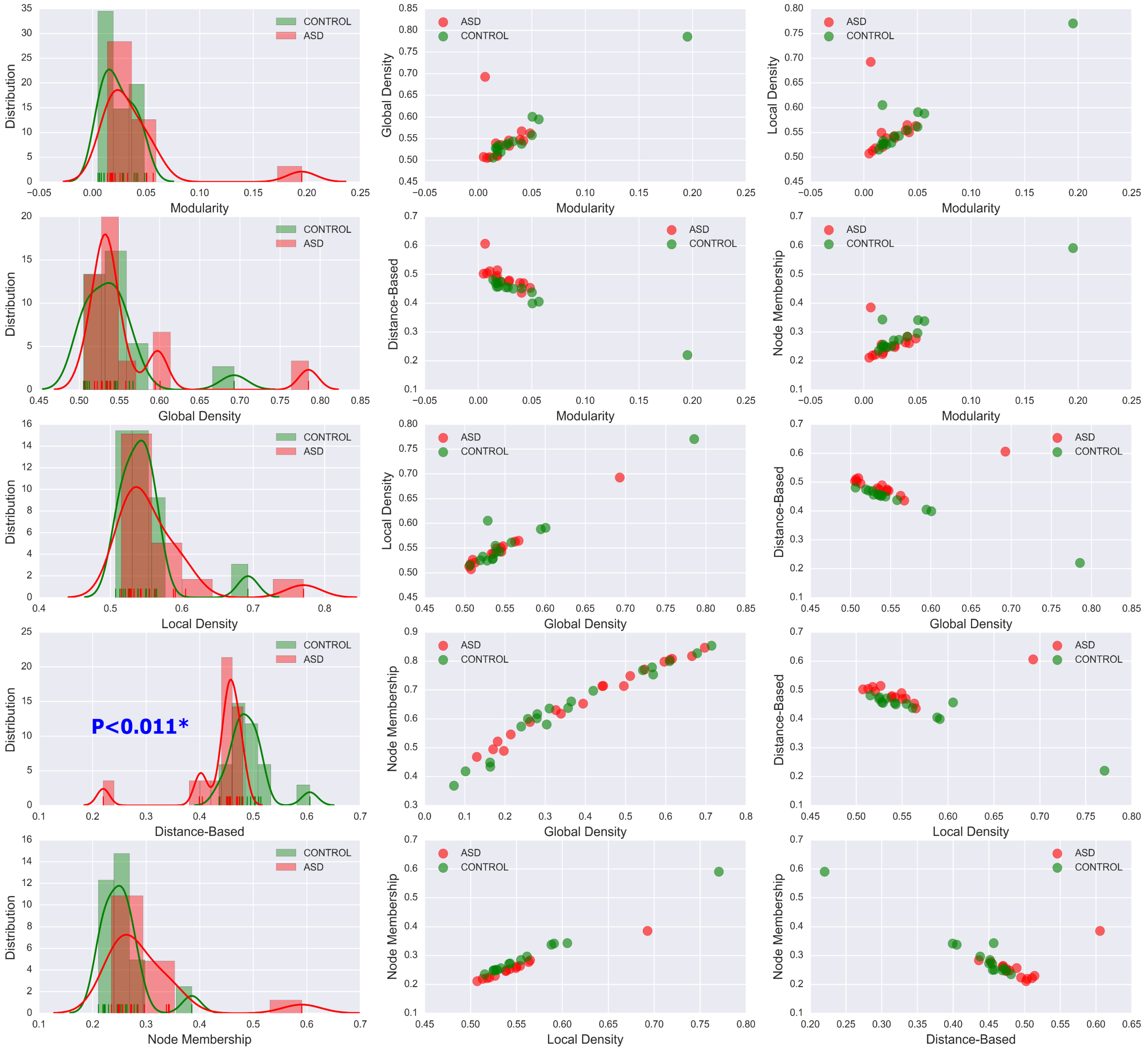

Figure S1: Left column: KDE plots of variations in the five community pattern metrics across subjects and clinical groups in the LV1 dataset at threshold T = 0.5 with a Gaussian kernel bandwidth of 0.02. These plots show significant differences in the distribution of community structure metrics between the two groups. Middle and right column: organisation of ASD and control group data visualised by scatter plots of all pairs of community pattern metrics.

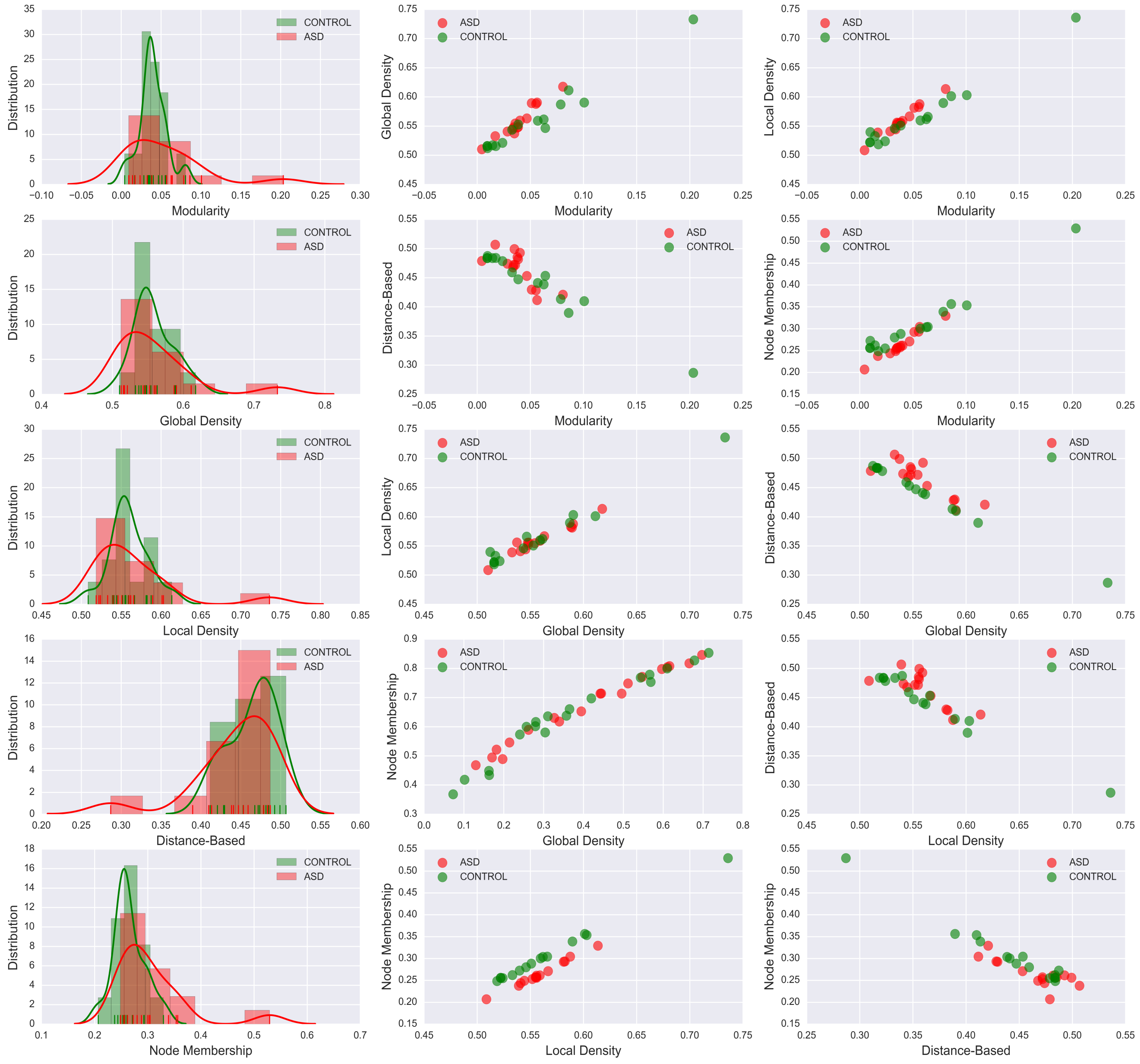

Figure S2: Left column: KDE plots of variations in the five community pattern metrics across subjects and clinical groups in the LV2 dataset at threshold T = 0.5 with a Gaussian kernel bandwidth of 0.02. Middle and right column: organisation of ASD and control group data visualised by scatter plots of all pairs of community pattern metrics.

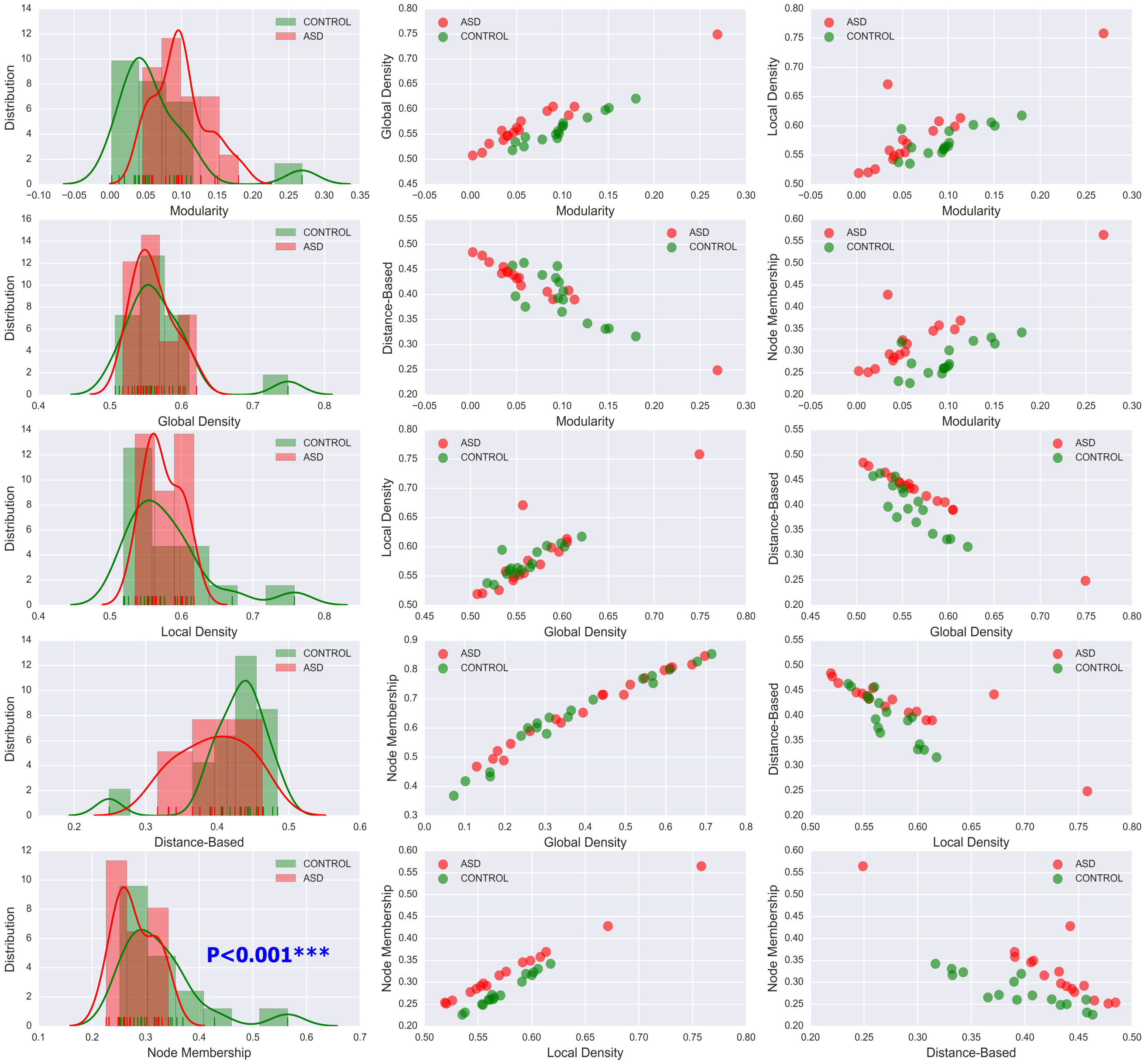

Figure S3: Left column: KDE plots of variations in the five community pattern metrics across subjects and clinical groups in the OLI dataset at threshold T = 0.5 with a Gaussian kernel bandwidth of 0.02. Middle and right column: organisation of ASD and control group data visualised by scatter plots of all pairs of community pattern metrics.

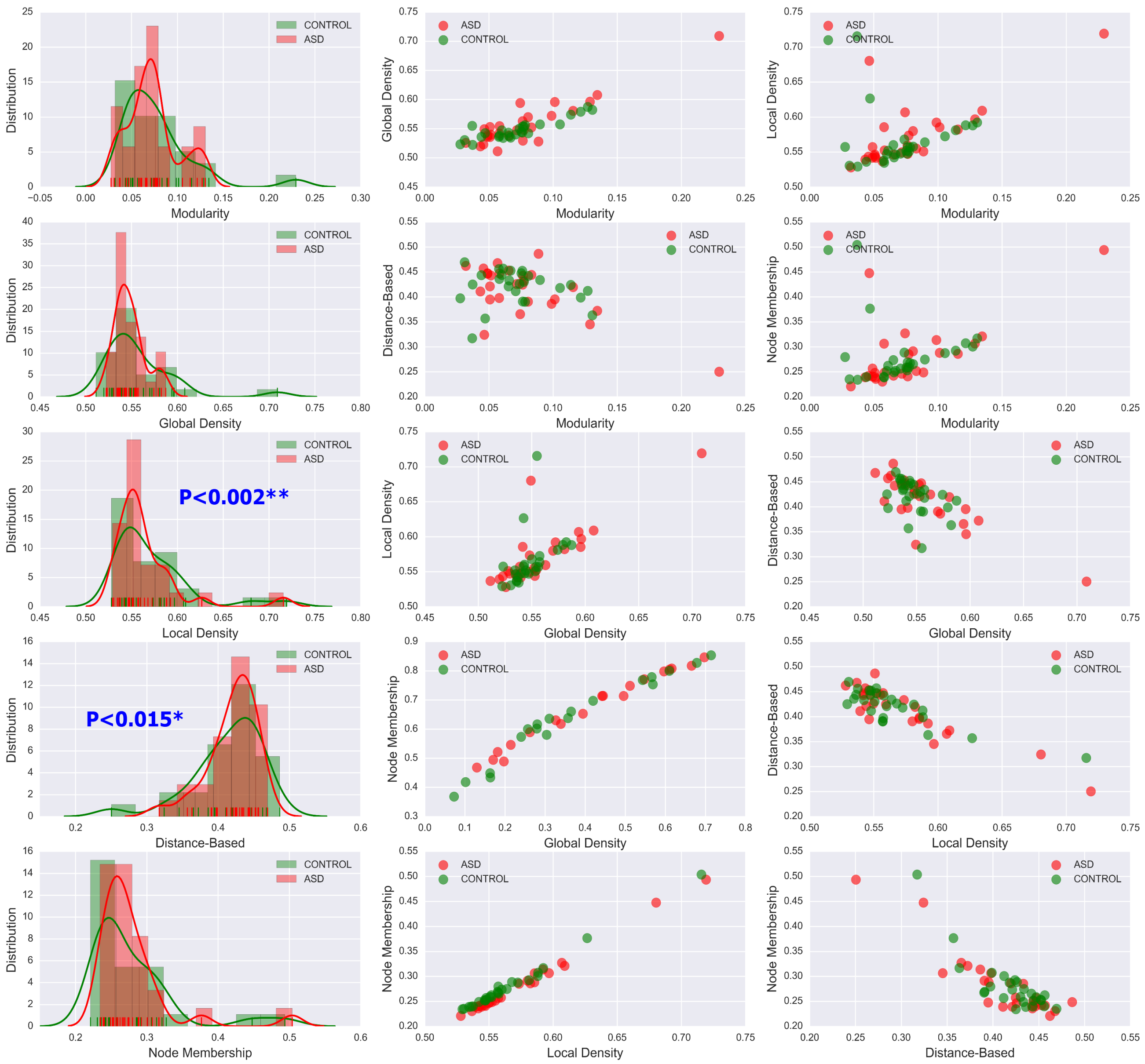

Figure S4: Left column: KDE plots of variations in the five community pattern metrics across subjects and clinical groups in the PIT dataset at threshold T = 0.5 with a Gaussian kernel bandwidth of 0.02. These plots show significant differences in the distribution of community structure metrics between the two groups. Middle and right column: organisation of ASD and control group data visualised by scatter plots of all pairs of community pattern metrics.

2 SUPPLEMENTARY MATERIAL FOR VALIDATION DATASETS

2.1 Supplementary Tables

Table S3: Validation datasets

	ASD		CONTROL		Age($\bar{x}\pm\sigma$)	Total
Dataset	M/F	Age	M/F	Age		$N = 214$
CMU	11/3	19-39	10/3	20-40	26.5±5.6	$n = 27$
KKI	16/4	8-12.5	20/8	8-12.8	10.0±1.3	$n = 48$
OHSU	12/0	8-15.2	14/0	8.2-11.9	10.8±1.87	$n = 26$
SBL	15/0	22-64	15/0	20-41	34.36±8.6	$n = 30$
SDSU	13/1	12.1-17.1	16/6	8.7-16.9	14.41±1.83	$n = 36$
TRI	22/0	12.0-25.9	25/0	12.0-25.7	17.17±3.63	$n = 47$

M = male, F = female, $\bar{x}$ = mean, σ = standard deviation.

Table S4: Imaging data acquisition parameters useful for preprocessing (validation datasets)

	CMU	KKI	OHSU	SBL	SDSU	TRI
Scanner	SIE VS MR B17	PHI A 3T	SIE MR B17	PHI 3T	GE 3T	PHI A 3T
Slice orientation	Transversal	Transversal	Transversal	Transversal	Interleaved	Transversal
#Slices	28	47	36	38	180	38
Voxel size (mm^3)	3×3×3	3×3×3	3.8×3.8×3.8	2.75×2.75×2.72	3.4×3.4×3.4	3×3×3.5
Resolution	64 × 64	84× 81	64 × 64	80×79	64×64	80×80
TR(ms)/TE(ms)	1500/30	2500 / 30	2500/30	2200 / 30	2000/30	2000/28
Flip angle (○)	73	75	90	80	90	90
Field of view (mm)	192	256	240	220	22	240

SIE VS MR B17 = SIEMENS MAGNETOM Verio syngo MR B17, PHI A 3T = 3 Tesla Philips Achieva,
SIE MR B17 = Siemens Magnetom TrioTim Syngo MR B17, PHI 3T = Philips Intera 3T, GE 3T = GE 3T MR750

Table S5: Rand Index values measuring the degree of agreement of community structures between control and ASD groups in validation data

T	CMU	KKI	OHSU	SBL	SDSU	TRI
0.1	0.59	0.69	0.78	0.89	0.95	0.70
0.2	0.64	0.64	0.76	0.83	0.82	0.68
0.3	0.76	0.66	0.70	0.64	0.74	0.64
0.4	0.78	0.58	0.68	0.65	0.69	0.59
0.5	0.56	0.76	0.77	0.71	0.70	0.68
0.6	0.66	0.87	0.81	0.90	0.94	0.74
0.7	0.86	0.89	0.92	0.99	0.98	0.88
0.8	0.98	0.99	1	0.99	0.99	0.99
0.9	1	1	1	1	1	1
$\bar{x}$	0.75	0.78	0.82	0.84	0.87	0.76
σ	0.15	0.14	0.11	0.13	0.12	0.14

T = sparsity threshold, $\bar{x}$ = mean, σ = standard deviation

Table S6: Results of Rand index testing (validation datasets)

Dataset	Mean within-CTR	Mean within-ASD	Mean of all within-group pairings in real data	Mean of all Within-group pairings with permuted labels	P-value real > permuted data
CMU (T = 3)	0.654	0.649	0.651	0.637	0.048
KKI (T = 3)	0.625	0.628	0.626	0.607	0.029
OHSU (T = 4)	0.548	0.528	0.538	0.497	0.041
SBL (T = 4)	0.498	0.489	0.493	0.486	0.026
SDSU (T = 2)	0.551	0.499	0.525	0.504	0.039
TRI (T = 6)	0.613	0.610	0.611	0.603	0.045
Multisite (T = 4)	0.600	0.597	0.593	0.586	0.031

P-values for mean group differences were estimated using a permutation test with $n = 50,000$ permutations.

2.2 Supplementary Figures

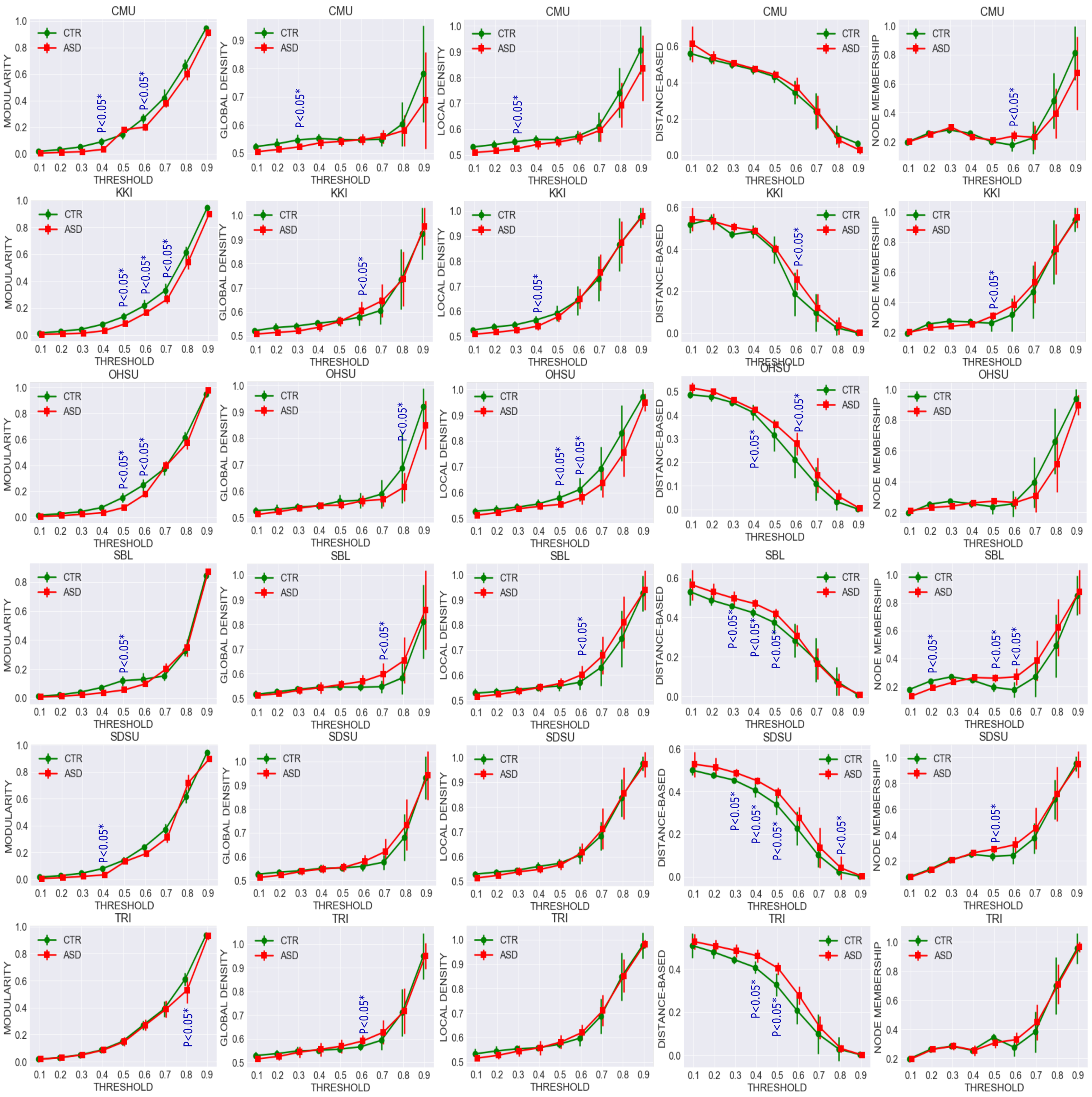

Figure S5: Comparing average and standard deviation of community pattern quality metrics between patients with ASD and controls in validation datasets. Community quality metrics were computed for each participant, and plots were created based on the average for patients with ASD and controls. Each row represents a dataset and each column one metric. Group statistical differences were analysed using the two-sample Kolmogorov-Smirnov test. Only significant FDR-corrected p-values are reported ($p < 0.05$).

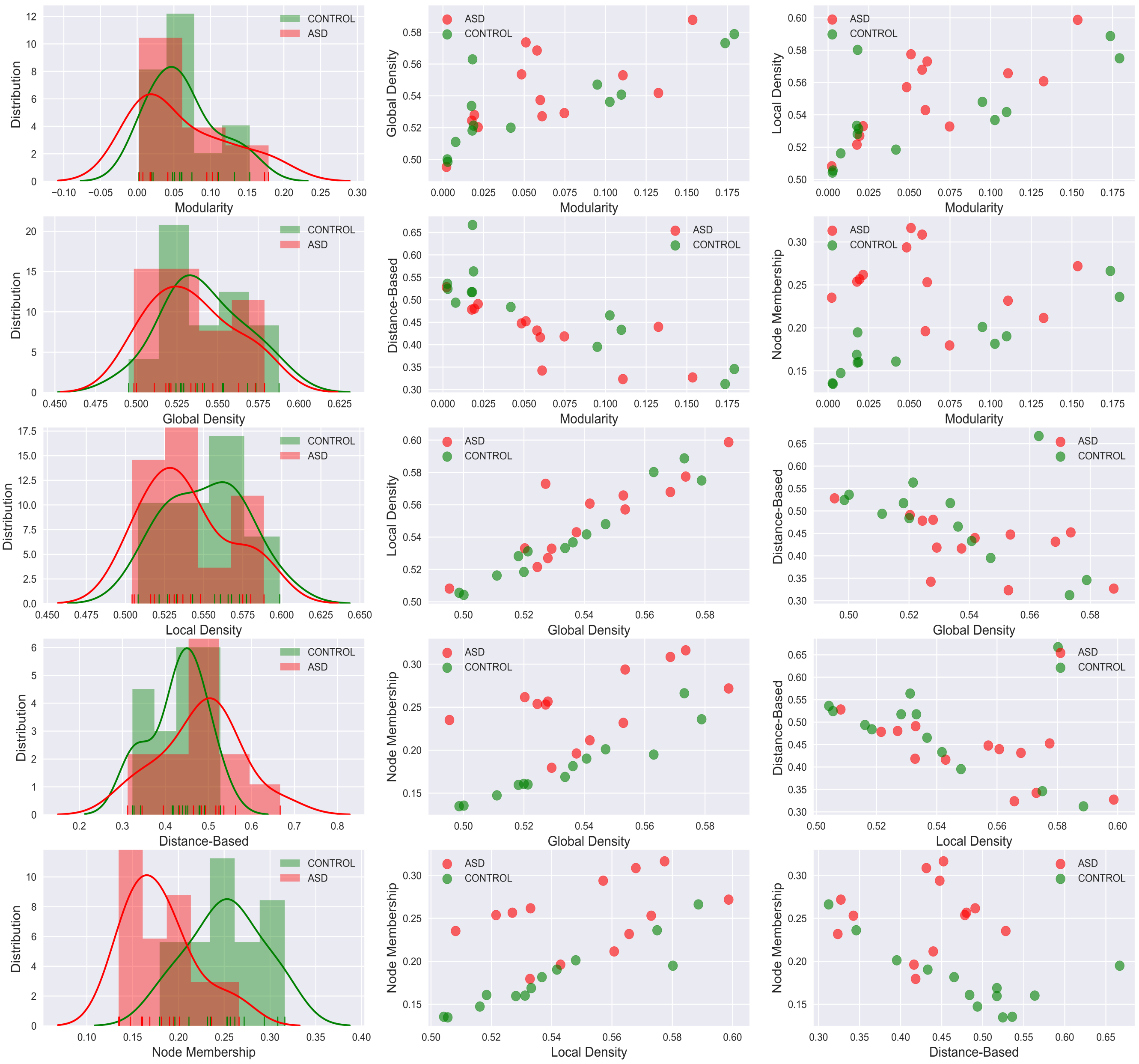

Figure S6: Left column: KDE plots of variations in the five community pattern metrics across subjects and clinical groups in the CMU dataset at threshold T = 0.3 with a Gaussian kernel bandwidth of 0.02. These plots show significant differences in the distribution of community structure metrics between the two groups. Middle and right column: organisation of ASD and control group data visualised by scatter plots of all pairs of community pattern metrics.

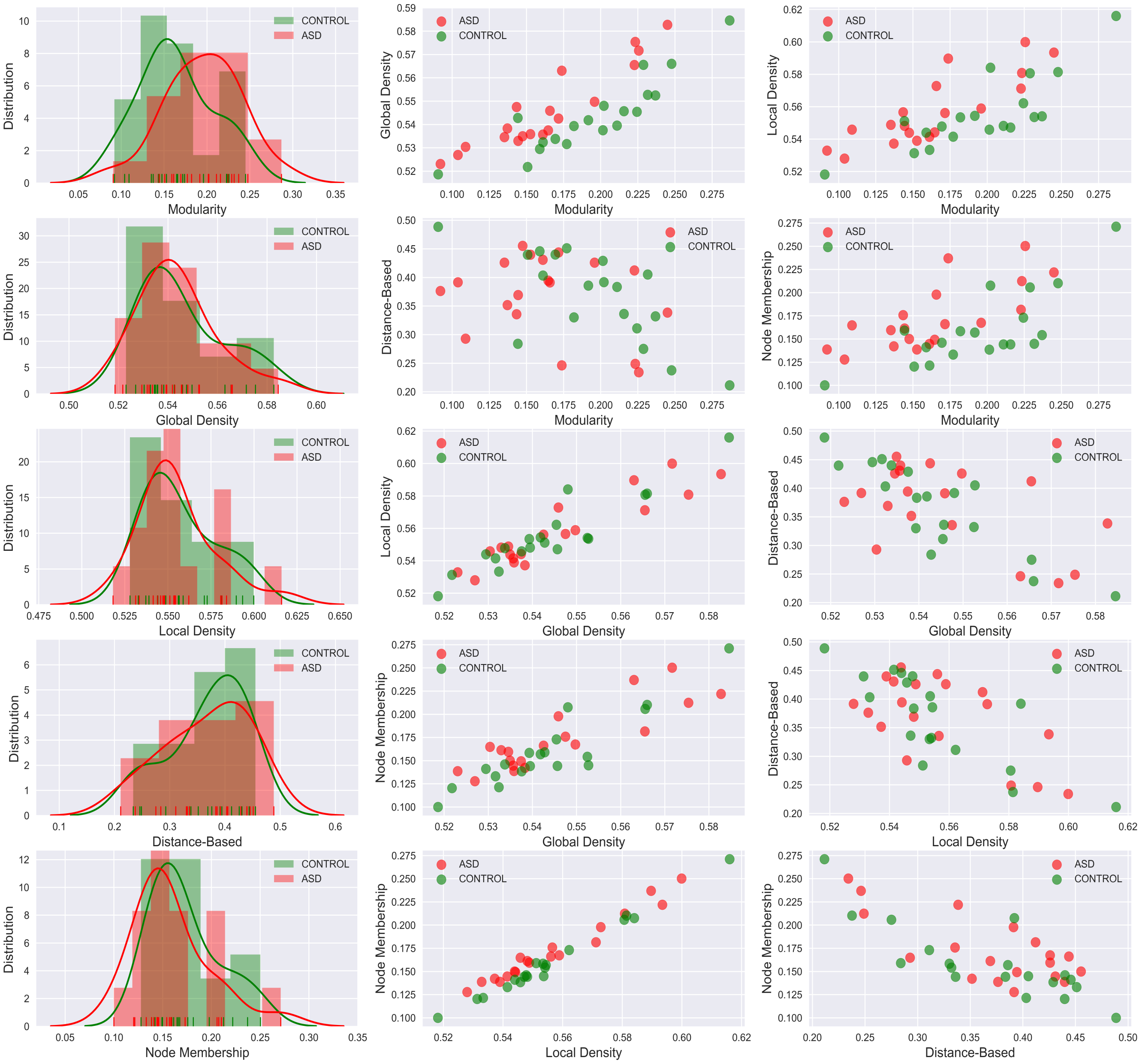

Figure S7: Left column: KDE plots of variations in the five community pattern metrics across subjects and clinical groups in the KKI dataset at threshold T = 0.3 with a Gaussian kernel bandwidth of 0.02. These plots show significant differences in the distribution of community structure metrics between the two groups. Middle and right column: organisation of ASD and control group data visualised by scatter plots of all pairs of community pattern metrics.

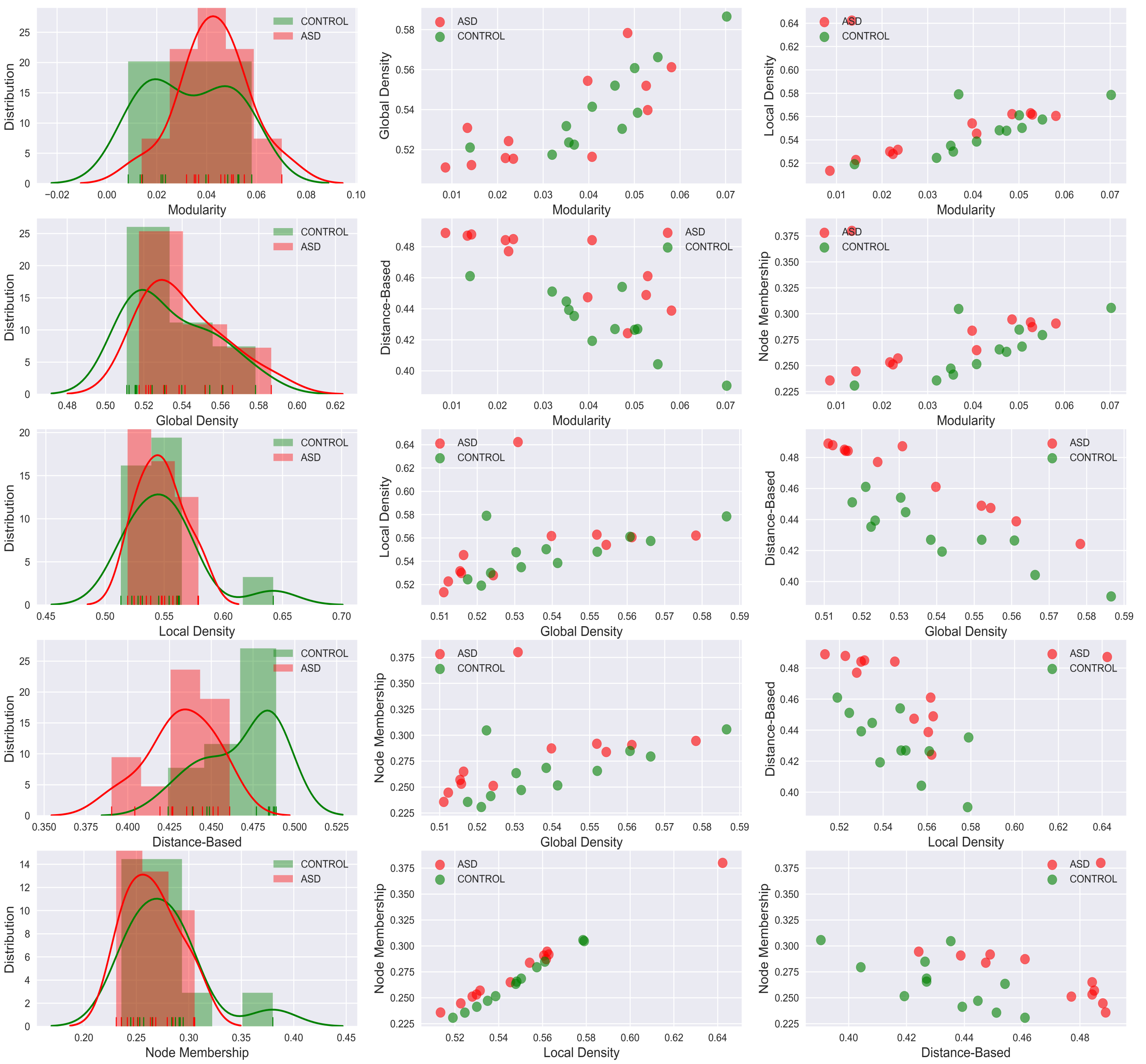

Figure S8: Left column: KDE plots of variations in the five community pattern metrics across subjects and clinical groups in the OHSU dataset at threshold T = 0.6 with a Gaussian kernel bandwidth of 0.02. These plots show significant differences in the distribution of community structure metrics between the two groups. Middle and right column: organisation of ASD and control group data visualised by scatter plots of all pairs of community pattern metrics.

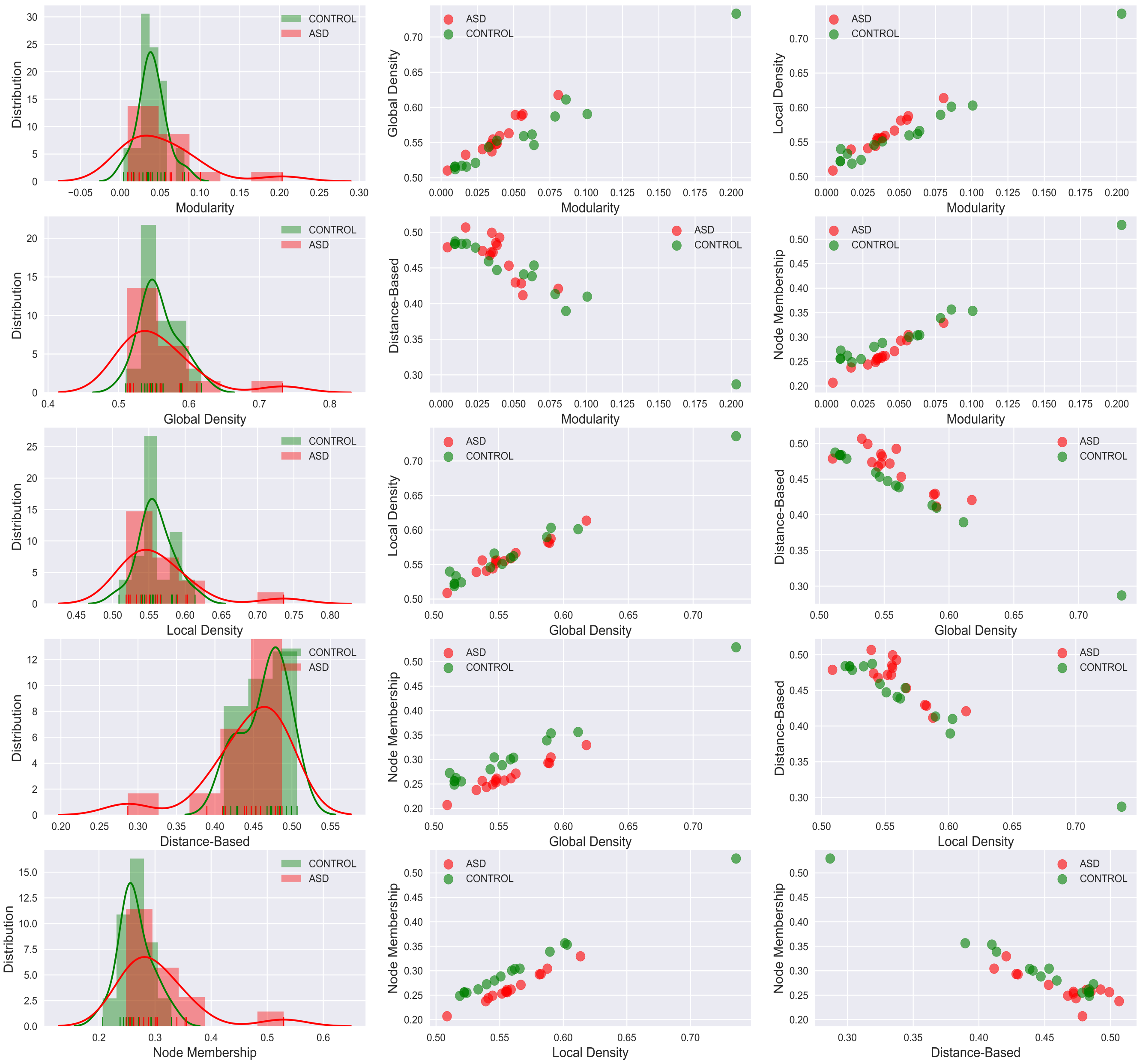

Figure S9: Left column: KDE plots of variations in the five community pattern metrics across subjects and clinical groups in the SBL dataset at threshold T = 0.7 with a Gaussian kernel bandwidth of 0.02. These plots show significant differences in the distribution of community structure metrics between the two groups. Middle and right column: organisation of ASD and control group data visualised by scatter plots of all pairs of community pattern metrics.

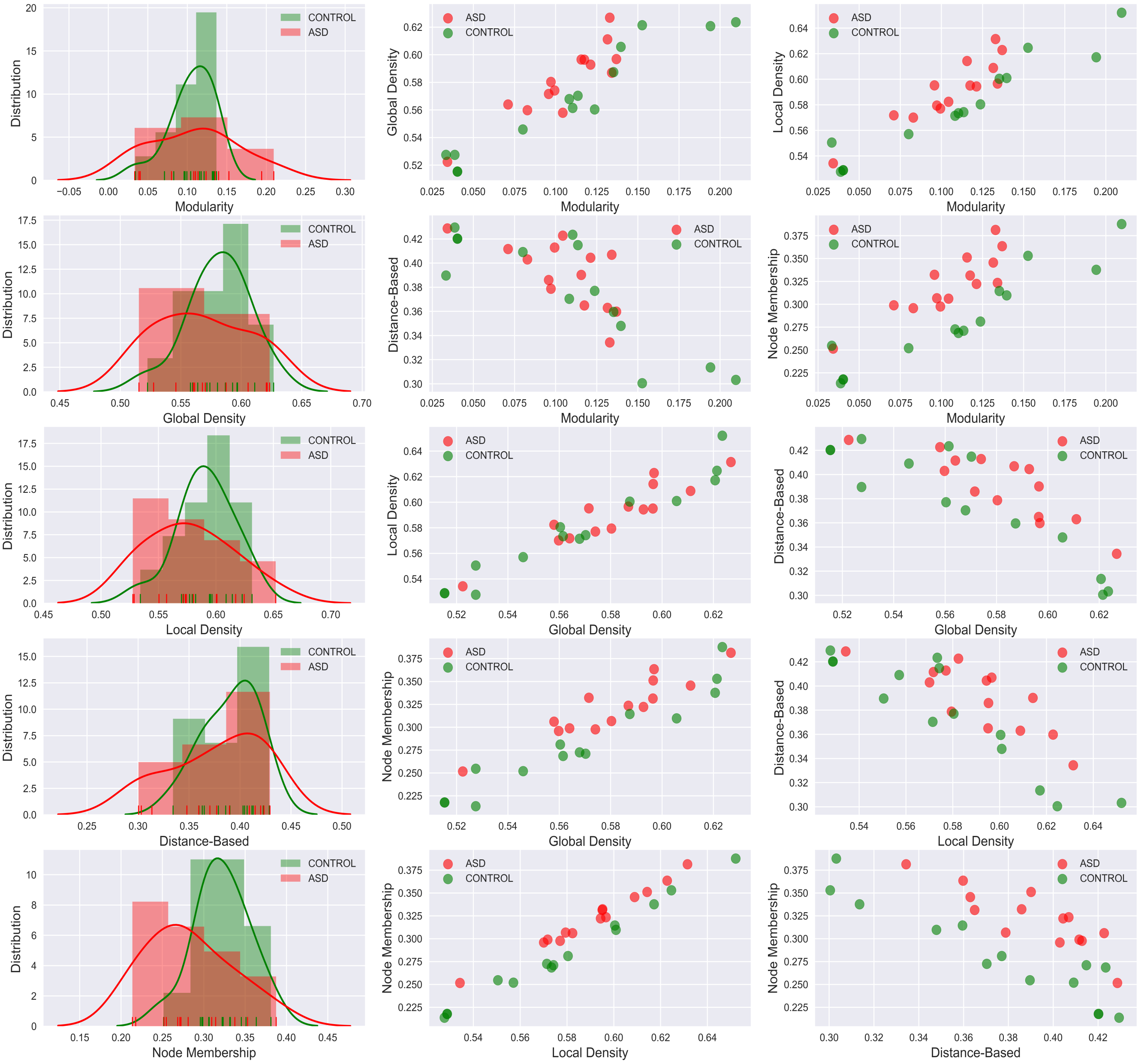

Figure S10: Left column: KDE plots of variations in the five community pattern metrics across subjects and clinical groups in the SDSU dataset at threshold T = 0.3 with a Gaussian kernel bandwidth of 0.02. These plots show significant differences in the distribution of community structure metrics between the two groups. Middle and right column: organisation of ASD and control group data visualised by scatter plots of all pairs of community pattern metrics.

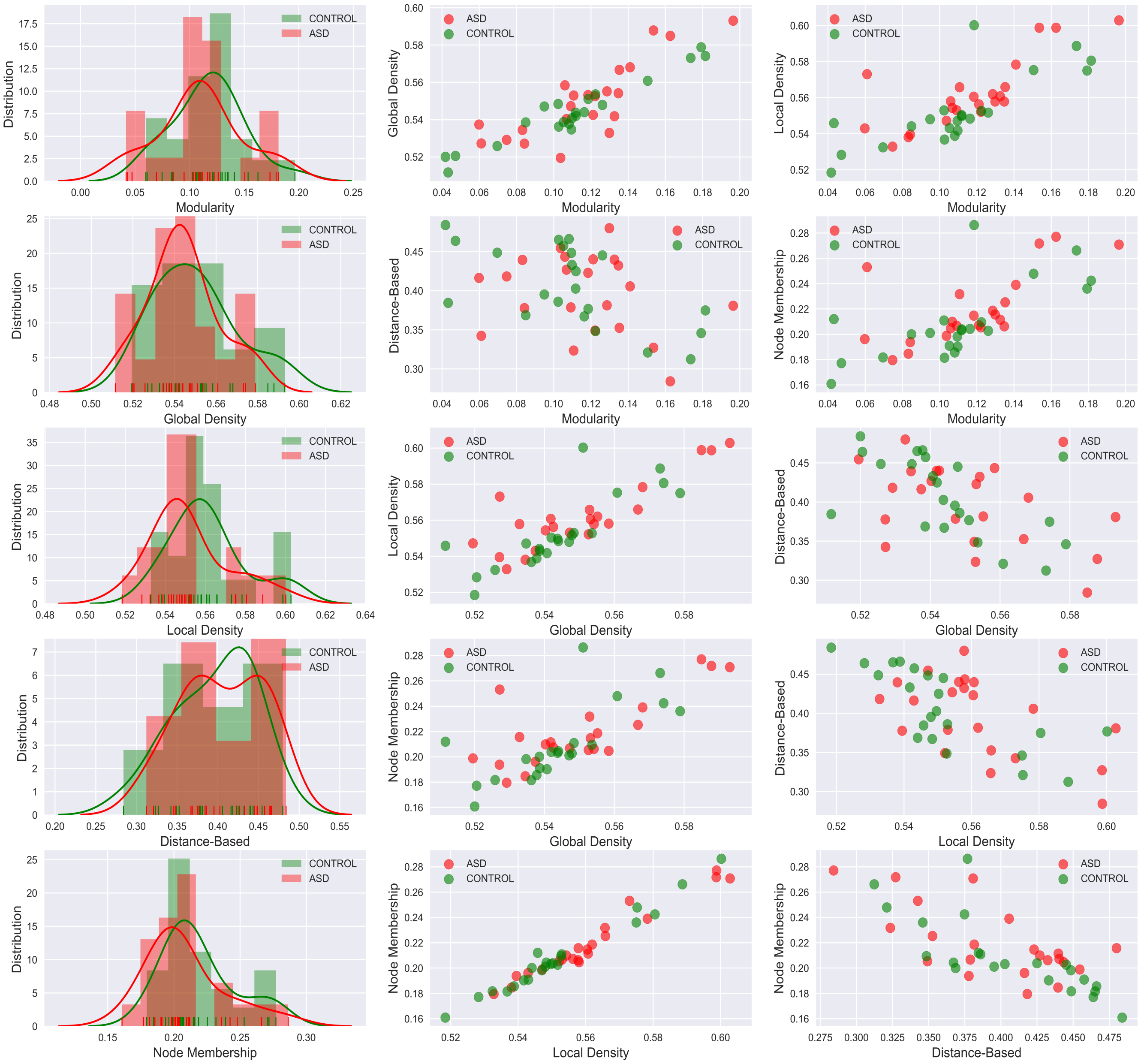

Figure S11: Left column: KDE plots of variations in the five community pattern metrics across subjects and clinical groups in the TRINITY dataset at threshold T = 0.8 with a Gaussian kernel bandwidth of 0.02. These plots show significant differences in the distribution of community structure metrics between the two groups. Middle and right column: organisation of ASD and control group data visualised by scatter plots of all pairs of community pattern metrics.
